# Supplementary material for: Evaluating cancer etiology and risk with a mathematical model of tumor evolution
Source: Nat Commun. 2022 Nov 24;13:7224. doi: 10.1038/s41467-022-34760-1 (PMC9700699; doi:10.1038/s41467-022-34760-1)
Supplement: Supplementary file 1 — Supplementary Information [file 41467_2022_34760_MOESM1_ESM.pdf]

SUPPLEMENTARY METHODS for

Evaluating cancer etiology and risk with a mathematical model of tumor evolution

Sophie Pénisson, Amaury Lambert and Cristian Tomasetti

In this document we shall denote by

- $P(t)$ the probability for an individual to get cancer by time t (or cumulative incidence rate),
- $I(t) = P'(t)$ the cancer incidence rate,
- $\lambda(t) = \frac{P'(t)}{1-P(t)}$ the cancer hazard rate,
- $\Lambda(t) = \int_0^t \lambda(s)\,ds$ the cumulative hazard function,

such that

$$P(t) = 1 - e^{-\Lambda(t)} = 1 - \frac{I(t)}{\lambda(t)}. \quad (1)$$

S1 A review of some key previous models

S1.1 Review

Contrary to typical descriptions, Charles and Luce-Clausen [1] were the ones to provide the first quantitative description of the multistage theory of cancer, as pointed out by Frank in [2]. They analyzed data from experiments on mice for which painting their skin with benzopyrene was associated with skin cancer. They assumed that cancer arose when both alleles of a single gene were knocked out. They reasoned that the probability of an allele being mutated in a cell after time t from initial exposure could be approximated by ut, where u is the mutation rate, i.e. the probability of that mutation occurring in a unit interval of time. They concluded that the probability of cancer by time t from the exposure was then given by $P(t) = Nu^2t^2$, where N is the total number of cells exposed to the carcinogen. The power of two in that expression is due to the fact that two independent mutational events have to happen for cancer to occur. Today we call these mutations "drivers" because they drive the process of tumorigenesis.

Nordling [3] then discovered a striking relationship between age and overall cancer incidence in humans by using a log-log plot. When the logarithm of age was plotted against the logarithm of cancer incidence at that age, an approximately linear relationship appeared to hold in each of the several countries examined. This suggested that the slope of that linear relationship could enable the estimation of the number of sequential rate-limiting steps n required to get to cancer. The idea behind is simple. By taking the derivative with respect to t of $P(t) = Nu^nt^n$ and then taking its logarithm yields $\ln I(t) = (n-1)\ln t + \ln(nu^nN)$. Thus, by estimating the slope $n-1$ of the linear relationship between cancer incidence $I(t)$ and age t in a log-log plot, we can estimate n. Nordling estimated $n = 7$, when combining the incidence of all cancer types together.

Inspired by Arley and Iversen [4], Armitage and Doll [5] made the insights of Nordling more precise by formulating the well-known multi-stage model of cancer incidence. They modeled the time X_{i+1} it takes to go from the arrival (or "hit") of the i-th cancer-driving mutation to the subsequent $i+1$-th hit, as an exponential random variable with (mutation) rate u_{i+1}. This implies that the probability of that driver mutation to occur by time t after the i-th hit is $1 - e^{-u_{i+1}t}$, which is approximately equal to $u_{i+1}t$, given that u_{i+1} is many orders of magnitude smaller than t^{-1}. By modeling these required driver mutations as independent events, it is relatively easy to show that their sum $X_1 + X_2 + \ldots + X_n$, a sum of exponentially distributed independent random variables, has probability density approximately equal to (for $u_it \ll 1$):

$$I(t) = Nu_1 \ldots u_n \frac{t^{n-1}}{(n-1)!}. \quad (2)$$

An important thing to notice is that no clonal expansion was included in this model. Clonal expansions, however, are critical for tumorigenesis and there is much evidence for clones of intermediate cells

(i.e. containing at least one driver mutation but not all the required ones for cancer) expanding into benign tumoral masses, like nodules found in the lungs or polyps in the colon. So while Armitage and Doll did not consider this important dynamics in [5], that changed in their 1957 model [6]. There, a new two-stage model assumed that after the first mutational hit, cells in this intermediate stage would grow exponentially until one of them would be hit by the second and final required driver mutation, yielding cancer. In that model, the incidence of cancer at time t from the initial exposure is given, for some constant κ, by:

$$I(t) = Nu_1\left(1 - e^{-\frac{u_2}{\kappa}\left(e^{\kappa t}-1\right)}\right). \tag{3}$$

The next step in the modeling of carcinogenesis was to include the different modes of stem cell divisions, as well as a death rate. The first well-known model of carcinogenesis to do this was the two-step clonal expansion model (TSCE) by Moolgavkar and Venzon [7], and the subsequent work by Moolgavkar with Knudson and Luebeck ([8], [9]) . In the TSCE model, symmetric cell division with rate α, cell differentiation or death with rate β, and asymmetric transformation with rate u_2, of intermediate cells yields that the hazard rate can be expressed analytically as (see also Heidenreich and Paretzke [10]):

$$\lambda(t) = \frac{Nu_1u_2\left(e^{(\gamma+2q)t}-1\right)}{q\left(e^{(\gamma+2q)t}+1\right)+\gamma}, \tag{4}$$

where $\gamma = \alpha - \beta - u_2$, and $q = \left(-\gamma + \sqrt{\gamma^2 + 4\alpha u_2}\right)/2$.

Finally, notable work was later produced especially by the group led by Nowak with Frank, Komorova, Michor, and Iwasa, as well as in work by Kimmel, and others. We choose to focus here on what we consider the most rigorous and complete model from a mathematical standpoint, specifically the work of Durrett and colleagues, who used branching processes and martingales. We will consider here specifically the model from Durrett and Moseley [11]. While this model has the limitation that it starts with the clonal expansion after the first hit, it is easy to approximate the general case as follows. Let $\lambda_i = b_i - d_i$ be the growth rate of type-i cells, $1 \leqslant i \leqslant n$, where b_i and d_i are the division and death rate of type-i cells, which mutate at rate u_{i+1} to cells of type $i+1$. Here, type n cells are cancer cells, while cells of type 0 are normal wild-type cells, and cells of type $1 \leqslant i \leqslant n-1$ are tumor cells at a non-cancerous intermediate stage. For each stem cell $1 \leqslant j \leqslant N$, let $T^{(j)}$ the waiting time for the first type-n cell in its lineage, assuming that n driver gene mutations are required. Assuming that the N stem cells have independent lines of descent, the probability to get cancer by time t is $P(t) = \mathbb{P}\left(\min_{j=1\ldots N} T^{(j)} \leqslant t\right) = 1 - (1 - F(t))^N$, where F is the cumulative distribution function of the random variable $T_0 + \tau_n$, with T_0 the waiting time to the first mutation in one healthy stem lineage (assumed to be exponentially distributed with parameter u_1), and τ_n the waiting time to the first type-n cell *after* the first mutation, as derived in [11]. Then $F(t) = \int_0^t u_1 e^{-u_1 s}\left(1 - \mathbb{P}(\tau_n > t-s)\right)ds$. Assuming that the approximations used in [11] can be applied here,

$$F(t) = \int_0^t \frac{u_1 e^{-u_1 s}\alpha\beta e^{\lambda_1(t-s)}}{1+\alpha\beta e^{\lambda_1(t-s)}}ds,$$

where $\alpha = \prod_{i=2}^n u_i^{\lambda_1/\lambda_{i-1}}$ and

$$\beta = \frac{b_1}{\lambda_1\lambda_{n-1}^{\lambda_1/\lambda_{n-1}}}\prod_{i=2}^{n-1}\left[\frac{\Gamma(1-\lambda_1/\lambda_i)\,\Gamma(1+\lambda_1/\lambda_i)\,b_i^{\lambda_1/\lambda_{i-1}}}{\lambda_{i-1}\lambda_i^{\lambda_1/\lambda_{i-1}}}\right]^{\lambda_1/\lambda_{i-1}}.$$

Since the cancer risk $P(t)$ is at most of the order of 10^{-1}, $F(t)$ is at most of the order of $\frac{1}{N}10^{-1}$. This implies that $|P(t) - NF(t)| \leqslant \frac{1}{2}(NF(t))^2 \ll P(t)$ and we can make the approximation $P(t) = NF(t)$. Using the inequalities $1 - x \leqslant (1+x)^{-1} \leqslant 1$ and $1 - x \leqslant e^{-x} \leqslant 1$ we obtain the following bounds

$$u_1\alpha\beta\int_0^t (1-u_1 s)\left(1-\alpha\beta e^{\lambda_1(t-s)}\right)e^{\lambda_1(t-s)}ds \leqslant F(t) \leqslant u_1\alpha\beta\int_0^t e^{\lambda_1(t-s)}ds.$$

The upper bound is $\frac{u_1\alpha\beta\left(e^{\lambda_1 t}-1\right)}{\lambda_1}$, and the lower bound can be written

$$\frac{u_1\alpha\beta\left(e^{\lambda_1 t}-1\right)}{\lambda_1}\left(1 - \frac{2\alpha\beta\lambda_1\left(e^{2\lambda_1 t}+tu_1-1\right)+u_1\left(e^{\lambda_1 t}-1\right)\left(4-\alpha\beta\left(1+e^{\lambda_1 t}\right)\right)-4u_1\lambda_1 t}{4\lambda_1}\right).$$

Since $u_1, \alpha, \beta \ll 1$, the term in the brackets is approximately 1, leading to the following approximation for the cancer risk

$$P(t) = Nu_1\alpha\beta\frac{e^{\lambda_1 t}-1}{\lambda_1}. \tag{5}$$

S1.2 Consistent conclusions

Note that since $P(t)$ is small (at most 13% for the most common cancer), the difference between $I(t)$ and $\lambda(t)$ (resp. between $P(t)$ and $\Lambda(t)$) is very small. Considering (2)-(5), we can conclude from all these key models as well as from our complete mathematical model for which the cancer risk is approximately $P(t) = \kappa N u^n t^n$ (formula (3) in the main text) the following properties:

- cancer incidence is a linear function of N, the population of cells at risk: multiplying N by a factor c will yield an incidence equal to $cI(t)$,
- cancer incidence is a non-linear function of t, the age of the individual; it is instead an exponential function or a power law,
- cancer incidence is a non-linear function of the successive mutation rates $u_1, \ldots, u_n$. However, assuming that each u_i is proportional to the individual mutation rate u, we conclude that the incidence is a power function of u, namely u^n where n is the number of stages or driver gene mutation hits. It ensues that multiplying u by a factor c, say because of an external factor, will yield an incidence equal to $c^n I(t)$. As a consequence, the ratio between the incidence curves of two groups of individuals, exposed (E) and unexposed (U) to a carcinogen, where group E has a mutation rate c times higher than U, will be given by

$$\frac{I_E(t)}{I_U(t)} = c^n.$$

S2 An analytical mechanistic model of tumor evolution

Our model relies on the analysis of three phenomena: the cell populations dynamics (Sections S2.1-S2.2), the appearance of driver mutations in these populations (Section S2.3), and the different effects of each mutation on the dynamics depending on the affected biological pathway (Section S2.4).

S2.1 Tissue stem cell population dynamics

Let N be the maximum number of stem cells that the tissue can sustain. The only assumption we make is that the stem cell population size N_t starts at conception time $-a_0$, and grows deterministically to size N (in finite time or asymptotically). As an example, we can assume that the deterministic process N_t consists of three phases, growing from 0 to δN during $\psi_0 = [-a_0, 0)$ (from conception to birth), from δN to N during $\psi_1 = [0, A)$ (youth), and having constant size N during $\psi_2 = [A, +\infty)$ (adulthood), where A and δ can be adjusted according to the tissue:

$$N_t = \begin{cases} a_0^{-1}\delta N t + \delta N, & t \in \psi_0, \\ A^{-1}(1-\delta) N t + \delta N, & t \in \psi_1, \\ N, & t \in \psi_2. \end{cases} \quad (6)$$

We may choose for instance $a_0 = 9$ months, $A = 15$ years and $\delta = 0.2$.

To account for the fact that the rates of the various cell division types (Fig. 5a) differ greatly depending on the tissue development phase, we assume that the proliferation rate b and asymmetric division probability p are time-dependent, which will in particular have an incidence on the mutation appearance rate (see Section S2.3). We can assume for instance that the rates are constant during each development phase:

$$b(t) = b_i, \; p(t) = p_i, \qquad t \in \psi_i, \quad (7)$$

with the asymmetric division rate kept constant across all phases, namely $b_i p_i =: \sigma$ for each i.

Remark. The values of p_i, b_i can be obtained as follows. Clearly, since $b_i p_i = \sigma$ is known, it is enough to compute $(1-p_i) b_i$. If one assumes that the tissue reaches size δN at birth, this means that $\ln(\delta N)/\ln 2$ self-renewal divisions occur during phase ψ_1, leading to $b_0(1-p_0) = \ln(\delta N)/a_0 \ln 2$. Similarly, growing from δN to N during ψ_1 while compensating for stem cell deaths implies that $b_1(1-p_1) = -\ln\delta/A\ln 2 + d$, where d is the death rate. Finally, during the homeostatic phase ψ_2 we can assume that self-renewal divisions compensate death, that is $b_2(1-p_2) = d$.

S2.2 Clonal stem cell population dynamics

We describe the evolution of any clonal population carrying a new driver mutation as follows (see Fig. 5b for a graphical summary). Note that all parameters mentioned hereafter might depend on the genotype of the clonal population, which is omitted in the notation for now. First, we assume that the clone has a certain carrying capacity C and that it starts with one single cell. Second, we assume that each stem cell renews itself at rate $(1-p)\,b$, asymmetrically differentiates at rate pb, symmetrically differentiates at rate d^s, or dies at rate d (Fig. 5a). We assume that the cell lineages initially evolve independently of each other, namely that the population grows like a branching process, until it eventually reaches a "survival" size εC at some random time τ (potentially infinite in case of extinction). By survival size we mean a size large enough such that it approximately ensures the survival of the population. The choice of ε is discussed in Section S2.5. According to the previous assumptions, its size initially follows a linear birth-and-death process with birth rate $(1-p)\,b$ and death rate d^s+d, hence with growth rate

$$r = (1-p)\,b - (d^s + d)\,. \tag{8}$$

Once the clone has reached size εC, we assume that its growth is deterministic and follows a logistic equation with carrying capacity C and growth rate (8). This is justified by the fact that for large populations the demographic stochasticity becomes negligible, hence it is reasonable to approximate a large population of size X_t by the solution to the logistic differential equation $\frac{dX_t}{dt} = r(1-\frac{X_t}{C})X_t$, where $r(1-\frac{X_t}{C})$ corresponds to the net increase rate per cell and per time unit in this population. Indeed, in order to take into account natural death as well as competition induced by the limited resources, we assume that the death rate per cell and per time unit is $d^s + d + r\frac{X_t}{C}$. Subtracting this death rate to the birth rate $(1-p)\,b$, we obtain the announced net increase rate per cell and per time unit. The overall growth of the clone at any time t is consequently given by

$$\begin{cases} X_t = \text{ birth-and-death process starting with one cell}, & 0 \leqslant t \leqslant \tau, \\ X_t = \dfrac{\varepsilon C e^{r(t-\tau)}}{1+\varepsilon\left(e^{r(t-\tau)}-1\right)}, & t \geqslant \tau. \end{cases}$$

An illustration of different population trajectories is given by Fig. 5b.

Some useful approximations

- Choosing ε adequately (see Section S2.5) enables to approximate the probability ρ of reaching the "survival" size εC for a cell population starting with one cell by the survival probability of the birth-and-death process, namely

$$\rho = \frac{r}{(1-p)\,b}. \tag{9}$$

- We also use on $[0,\tau]$, when it is relevant, the approximation of the birth-and-death process by its limiting distribution in the supercritical case, deduced from branching process theory. Denoting by $\Omega_0 = \{\lim_t X_t = 0\}$ the extinction set of the process, it is known that $\lim_t e^{-rt}X_t = W$ almost surely, where $\mathbb{P}(W=0) = \mathbb{P}(\Omega_0) = \frac{d^s+d}{(1-p)b}$ and $W \mid \Omega_0^c$ is exponentially distributed with parameter ρ. This implies in particular that the probability of reaching size εC before time t can be approximated by

$$\mathbb{P}(\tau \leqslant t) = \mathbb{P}(X_t \geqslant \varepsilon C) \approx \mathbb{P}\left(W \geqslant e^{-rt}\varepsilon C \mid \Omega_0^c\right) = e^{-\rho\varepsilon C e^{-rt}}.$$

Therefore, the latency period τ (conditional on reaching size εC in finite time) is of the order of $\frac{1}{r}\ln(\rho\varepsilon C)$. More precisely, it can be written as

$$\tau = \frac{1}{r}\left(\ln(\rho\varepsilon C) + \tilde{\tau}\right) \tag{10}$$

where $\tilde{\tau}$ is a standard Gumbel random variable, i.e. with density $\tilde{f}(t) = e^{-\left(t+e^{-t}\right)}$ on $\mathbb{R}$ and $\mathbb{E}(\tilde{\tau}) = \gamma \approx 0.58$ (Euler's constant). In particular, $\tilde{\tau}$ is negligible compared to $\ln(\rho\varepsilon C)$ if $\rho\varepsilon C$ gets large. The density of τ on $\mathbb{R}$ is immediately deduced from (10) as

$$f(t) = r\tilde{f}\left(rt - \ln(\rho\varepsilon C)\right). \tag{11}$$

We show in Section S2.5 that if ε is chosen appropriately, the probability for τ to be negative is close to 0, in which case (11) can be used as a density for the positive random variable τ.

- In Section S2.4 we will make use of the fact that the death rates d^s and d are often small compared to $(1-p)\,b$, leading to the fact that an increase of $(1-p)\,b$ approximately translates into an increase of r of the same order of magnitude, that is for $\alpha > 0$

$$(1+\alpha)\,(1-p)\,b - (d^s + d) \approx (1+\alpha)\,r. \tag{12}$$

S2.3 Appearance of driver mutations

Let u be the driver mutation probability per cell division in a given population or subpopulation. We deduce from Section S2.2 that the driver mutation rate per cell and per time unit due to errors during asymmetric (resp. symmetric) division is pub (resp. $2\,(1-p)\,ub$), hence that the driver mutation rate per cell and per time unit in a clone is equal to

$$\nu = (2-p)\,ub. \tag{13}$$

Since p and b in the tissue cell population are time-dependent (see Section S2.1), the driver mutation rate per cell and per time unit in the tissue population is time-dependent equal to $\nu\,(t) = (2 - p\,(t))\,ub\,(t)$. In the particular case of constant parameters p and b during each tissue development phase, it becomes

$$\nu\,(t) = \nu_i = (2-p_i)\,ub_i, \quad t \in \psi_i. \tag{14}$$

The appearance rate of any driver mutation in the tissue stem cell population is thus $\nu\,(t)\,N_t$, where N_t is the deterministic tissue population size (Section S2.1), while the appearance rate on a clone with initial size εC is $\nu\,(t)\,\frac{\varepsilon C e^{rt}}{1+\varepsilon(e^{rt}-1)}$.

S2.4 Effect of a driver mutation on the population growth and mutation parameters

Let $\mathcal{M}$ be the set of the three possible driver gene mutation types $\mathcal{M} = \{S, F, M\}$, where S stands for the mutations affecting the cell survival process, F for those affecting the cell fate, and M for those affecting the genomic maintenance. Let us consider a driver mutation $V \in \mathcal{M}$ appearing on a cell population with a given genotype and genotype-dependent growth and mutation parameters (b, p, u) (we omit here in the notation the dependence on the genotype). We assume that depending on its type, a driver mutation V has a direct effect on these parameters. Moreover, if the clone carrying V appears on the tissue cell population, this effect also depends on the clone appearance time, for instance on the tissue development phase ψ_i at that time (see Section S2.1). The effects of V on the growth and mutation parameters b, p, u of the population, which in turn translate into effects on the parameters r and C, are detailed below and summarized in Table S1. This provides in particular the multiplicative effect γ_V of V on νC, that is

$$\nu_V C_V = \gamma_V \nu C. \tag{15}$$

Note that even though only the stem cells are counted, the carrying capacity C of the stem cell population should take into account the variation in the number of differentiated cells coming from the normal development or from the increase in fitness of some driver mutations. It seems indeed reasonable to assume a common competition intensity between both cell types, inversely proportional to the total carrying capacity of the clone

$$C\,(1+\lambda)\,, \tag{16}$$

where λ is the ratio between the differentiated cells and the stem cells carrying capacities in the clonal population.

Effect of a S mutation The appearance of a S mutation induces an increase of the division rate, namely $b_S = (1+\alpha)\,b$, which leads to $\nu_S = (1+\alpha)\,\nu$. This increase in b results in an increase of the total carrying capacity (16), which becomes $(1+\alpha)\,C_S\,(1+\lambda_S)$. Yet it does not affect the relative dynamics between the differentiated and stem cell population, hence $\lambda_S = \lambda$, leading to $C_S = (1+\alpha)\,C$. Finally, by (12), the growth rate of the subsequent clonal population is then $r_S \approx (1+\alpha)\,r$. Note that if S appears on the tissue cell population and the clone reaches its survival size at time t, the values of p, b, r and ν in what precedes are taken at time t.

Effect of a F mutation The appearance of a F mutation induces a decrease of the asymmetric division probability, say $p_F = (1-\beta)\,p$. This decrease biases the asymptotic proportions of the stem cells versus the differentiated cells, namely $\lambda_F = (1-\beta)\,\lambda$. However, the total carrying capacity (16) of the clonal population remains unchanged, namely $C\,(1+\lambda) = C_F\,(1+\lambda_F)$, leading to

$$C_F = \left(1 + \frac{\beta\lambda}{1+(1-\beta)\,\lambda}\right) C.$$

Finally, by (12),

$$r_F \approx \left(1+\beta\frac{p}{1-p}\right) r, \quad \nu_F = \left(1+\beta\frac{p}{2-p}\right)\nu.$$

Again, if F appears on the tissue cell population and the clone reaches its survival size at time t, the values of p, b, r and ν are taken at time t.

Effect of a M mutation The appearance of a M mutation induces an increase of the mutation probability, namely $u_M = (1+\delta)\,u$. The carrying capacity thus remains unchanged, $C_M = C$, and $\nu_M = (1+\delta)\,\nu$. If M appears on the tissue cell population and the clone reaches its survival size at time t, the values of p, b, r and ν are taken at time t.

growth and mutation parameters of the V-population		mutation type $V=S$	$V=F$	$V=M$
birth rate	b_V	$(1+\alpha)\,b$	b	b
asymmetric division prob.	p_V	p	$(1-\beta)\,p$	p
mutation prob.	u_V	u	u	$(1+\delta)\,u$
growth rate	r_V	$(1+\alpha)\,r$	$\left(1+\beta\frac{p}{1-p}\right) r$	r
carrying capacity	C_V	$(1+\alpha)\,C$	$\left(1+\frac{\beta\lambda}{1+(1-\beta)\lambda}\right) C$	C
mutation appearance rate	ν_V	$(1+\alpha)\,\nu$	$\left(1+\beta\frac{p}{2-p}\right)\nu$	$(1+\delta)\,\nu$
multiplicative effect on νC	γ_V	$(1+\alpha)^2$	$\left(1+\beta\frac{p}{2-p}\right)\left(1+\frac{\beta\lambda}{1+(1-\beta)\lambda}\right)$	$1+\delta$

Table S1: **Effect of a driver mutation** $V \in \{S, F, M\}$ on a cell population with parameters b, p, u. If the mutation appears on the tissue cell population, the parameters b, p, r, ν are taken at the time when the clone carrying V reaches a "survival" size.

S2.5 Choice of the threshold in the analytical model

The constant ε introduced in Section S2.2 should be chosen in such a way that:

(i) The survival probability of the birth-and-death process is close to the probability of reaching size εC. Using the strong convergence $\lim_t e^{-rt}X_t = W$ exposed in Section S2.2, the fact that X_t conditionally on Ω_0 behaves like a subcritical birth-and-death process $\tilde{X}_t$ with birth rate $d+d^s$ and death rate $(1-p)\,b$, and Doob's martingale inequality applied to the martingale $e^{rt}\tilde{X}_t$, we obtain

$$\begin{aligned}
\mathbb{P}\,(\exists t:\, X_t > \varepsilon C) &= \frac{d^s+d}{(1-p)\,b}\mathbb{P}\,(\exists t:\, X_t > \varepsilon C \mid \Omega_0) + \rho\mathbb{P}\,(\exists t:\, X_t > \varepsilon C \mid \Omega_0^c) \\
&= \frac{d^s+d}{(1-p)\,b}\mathbb{P}\left(\exists t:\, \tilde{X}_t > \varepsilon C\right) + \rho \\
&\leqslant \mathbb{P}\left(\exists t:\, \tilde{X}_t > \varepsilon C\right) + \rho \\
&\leqslant \lim_n \mathbb{P}\left(\max_{t\in[0,n]} e^{rt}\tilde{X}_t \geqslant \varepsilon C\right) + \rho \\
&\leqslant \lim_n \frac{\mathbb{E}\left(e^{rn}\tilde{X}_n\right)}{\varepsilon C} + \rho \\
&= \frac{1}{\varepsilon C} + \rho.
\end{aligned}$$

Hence for εC large enough ($\varepsilon C \gg \rho^{-1}$), this probability is close to the survival probability ρ, which justifies approximation (9).

(ii) The approximation of X_t by $e^{rt}W$ remains valid when X_t becomes close to εC, which requires W to have negligible probability to be larger than εC. We have $\mathbb{P}(W \geqslant \varepsilon C \mid \Omega_0^c) = e^{-\rho\varepsilon C}$. For εC large enough ($\varepsilon C \gg \rho^{-1}$), this probability is close to 0, which justifies approximation (11) for the probability density function of the latency period τ.

(iii) It is reasonable to assume that no mutation occurs during the time-interval $[0, \tau]$. Indeed, deducing from (10) $\mathbb{E}(\tau) = \frac{1}{r}(\ln(\rho\varepsilon C) + \gamma)$, we obtain by Jensen's inequality

$$\begin{aligned}\mathbb{P}(\text{mutation during } [0,\tau]) &= \mathbb{E}\left[\mathbb{P}\left(\text{mutation during } [0,\tau] \mid (X_t)_{t\in[0,\tau]}\right)\right] \\ &= 1 - \mathbb{E}\left(e^{-\nu\int_0^\tau X_t dt}\right) \\ &\leqslant 1 - \mathbb{E}\left(e^{-\nu\varepsilon C\tau}\right) \\ &\leqslant 1 - e^{-\nu\varepsilon C\mathbb{E}(\tau)} \\ &= 1 - e^{-\frac{(2-p)u\varepsilon C}{(1-p)\rho}(\ln(\rho\varepsilon C)+\gamma)},\end{aligned}$$

which for εC small enough ($\varepsilon C(\ln(\rho\varepsilon C) + \gamma) \ll \frac{(1-p)\rho}{(2-p)u}$) is close to 0 (or even negative).

For instance, for clonal populations for which the death rate $d + d_s$ is small compared to the birth rate $(1-p)b$, say $d + d_s \leqslant 0.1(1-p)b$ (implying $\rho \in [0.9, 1]$), for which the driver mutation rate u is smaller than 10^{-6}, and the asymmetric division probability p is smaller than 0.95 (which are all reasonable assumptions, see end of Section S3.2), then choosing $\varepsilon C = 10^2$ ensures that the previous approximations hold true with an error bound of the order of 10^{-2}, namely

$$|\mathbb{P}(\exists t:\ X_t > \varepsilon C) - \rho| \leqslant 10^{-2},$$

$$\mathbb{P}(W \geqslant \varepsilon C \mid \Omega_0^c) \leqslant 10^{-2},$$

$$\mathbb{P}(\text{mutation during } [0,\tau]) \leqslant 10^{-2}.$$

S3 The timing of cancer

S3.1 Analytical expression of cancer risk

For a given tissue, let $\mathcal{C}$ be the set of the associated "cancer" genotypes. For example, $\mathcal{C}$ can be the set of genotypes carrying at least n driver mutations for some fixed integer n, of which at least one is S-type and one is F-type. We assume that an individual gets a given cancer once it develops a clone with genotype $G \in \mathcal{C}$ of non-negligible size, i.e. of size εC_G (Fig. S1).

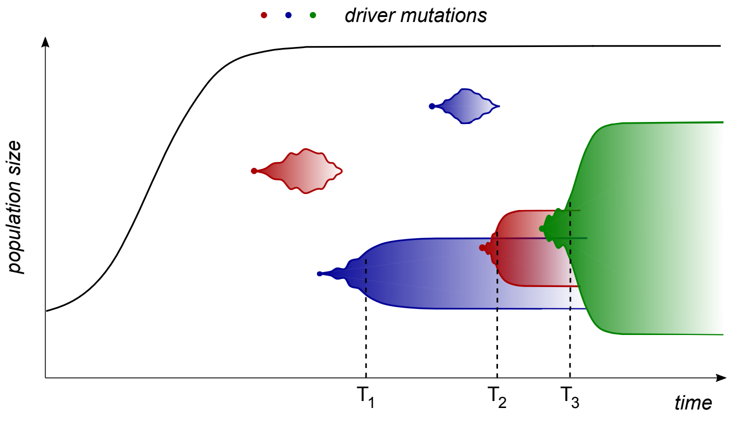

Figure S1: **Accumulation of three driver gene mutations**. The tissue gets cancer at time T_3.

The cancer risk $P(t)$ of an individual with a mutation-free genotype at conception, namely its probability to get cancer before age t, can be expressed iteratively thanks to the following quantity. For any

genotype G carrying at least one driver mutation, we define $P_G(t)$ as the probability for a G-clone of size εC_G to lead to cancer in less than t time units. Assuming independence of the clonal populations, we deduce from point process theory that $P_G(t) = 1$ if $G \in \mathcal{C}$, while if $G \notin \mathcal{C}$,

$$P_G(t) = 1 - \exp\left(-\sum_{V\in\mathcal{M}} \int_0^t \int_0^s \nu_G X_v^G \pi_{GV} \rho_{GV} f_{GV}(s-v) P_{GV}(t-s)\, dvds\right). \tag{17}$$

The variable v corresponds to the appearance time of an additional mutation V in the G-population, and $s \geqslant v$ to the time at which the GV-clone reaches size εC_{GV}. The exponential term in (17) is then the probability that no such mutation leading to cancer in less than $t - s$ units occurs. More precisely,

- ν_G is the appearance rate per cell per time unit (13) of a driver mutation in the G-population with deterministic size $X_t^G = \frac{\varepsilon C_G e^{r_G t}}{1+\varepsilon(e^{r_G t}-1)}$, $t \geqslant 0$.
- for any $V \in \mathcal{M}$, π_{GV} is the probability that a driver mutation appearing in the G-population is of type V (with $\pi_{GS} + \pi_{GF} + \pi_{GM} = 1$),
- ρ_{GV} is the probability (9) for a GV-clone of reaching size εC_{GV},
- f_{GV} is the latency period distribution (11) to reach this size, conditional on reaching it in finite time,
- and $P_{GV}(s)$ is by definition the probability for a GV-clone with initial size εC_{GV} to lead to cancer in less than s time units, which can in turn be computed thanks to (17).

The growth and mutation parameters of the GV-clone needed to compute (9), (11) and iterate (17) depend on the genotype G and on the type of the mutation V, as described in Table S1.

The cancer risk $P(t)$ is then computed in a similar manner by considering the appearance of clones in the tissue population. The only difference is that time starts at conception $-a_0$ and that the mutation rate in the tissue population is time-dependent. In addition, the parameters required to compute the survival probability and cancer risk of a newly arising clonal population depend on the appearance time of this clone, more precisely on the time when it reaches a survival size. Assuming for instance constant division rates on each development phase ψ_i of the tissue as described in Section S2.1, we obtain for any $t \geqslant -a_0$,

$$P(t) = 1 - \exp\left(-\sum_{V\in\mathcal{M}} \sum_{i=0}^{2} \int_{\psi_i} \int_{-a_0}^{s} \nu_i N_v \pi_V \rho_V^i f_V^i(s-v) P_V^i(t-s) \mathbf{1}_{s\leqslant t} dvds\right),$$

where ν_i is given by (14) , N_v is the deterministic tissue population size (Section S2.1), and ρ_V^i, f_V^i, P_V^i depend on parameters $p_V^i, b_V^i, \ldots$ which correspond to the effects of mutation V on the tissue population parameters $p_i, b_i, \ldots$ during phase ψ_i, described in Section S2.4. Note that in particular the cumulative cancer hazard function defined in (1) is given by

$$\Lambda(t) = \sum_{V\in\mathcal{M}} \sum_{i=0}^{2} \int_{\psi_i} \int_{-a_0}^{s} \nu_i N_v \pi_V \rho_V^i f_V^i(s-v) P_V^i(t-s) \mathbf{1}_{s\leqslant t} dvds. \tag{18}$$

Here again, v corresponds to the appearance time of a mutation V, and $s \geqslant v$ to the time at which the V-clone reaches size εC_V, where we differentiate whether $s \in \psi_0$, ψ_1 or ψ_2.

S3.2 Simplified expression of the cancer time distribution

In order to isolate the effect of u, b and N on the cancer risk $P(t)$, we make the following simplifying approximations in our model. We assume that any newly appearing mutation gives rise to a clone of size equal to its carrying capacity (i.e. with no initial random phase nor growth phase), that the tissue population grows linearly according to (6) with $\delta = 1$ (i.e. reaches its full size N at birth), and that the mutation division rate is constant across youth and adulthood $\nu_1 = \nu_2 = \nu = (2-p)\,ub$, while $\nu_0 = (2-p_0)\,ub_0$ between conception and birth (7). We know from (15) that for any genotype $G = V_1 \cdots V_k$ for which the first mutation V_1 appears before birth, $\nu_G C_G = \gamma_{V_1} \cdots \gamma_{V_k} \nu_0 C$, while if V_1 appears after birth, $\nu_G C_G = \gamma_{V_1} \ldots \gamma_{V_k} \nu C$. Finally, we assume here for simplicity that the set of cancer genotypes $\mathcal{C}$ associated with the tissue consists of genotypes with exactly n driver mutations, for some

fixed integer n. For instance, $\mathcal{C}$ can be the set of genotypes with $n = 3$ driver mutations, of which at least one is of type S and one is of type F. We define the constant κ_n as the sum over all possible cancer genotypes of cumulated multiplicative effects (15) of the first $n-1$ mutations, weighted by the probability of each genotype:

$$\kappa_n = \sum_{V_1 \cdots V_n \in \mathcal{C}} \gamma_{V_1}^{n-1} \gamma_{V_2}^{n-2} \dots \gamma_{V_{n-1}} \pi_{V_1} \pi_{V_1 V_2} \dots \pi_{V_1 \dots V_n}. \tag{19}$$

It ensues in particular that $\kappa_n C^{n-1} \nu^{n-1}$ (resp. $\kappa_n C^{n-1} \nu_0^{n-1}$) corresponds to the average over all possible cancer genotypes $V_1 \cdots V_n$ of the product $\nu_{V_1} C_{V_1} \dots \nu_{V_1 \dots V_{n-1}} C_{V_1 \dots V_{n-1}}$, if V_1 occurs after birth (resp. before birth). Under these assumptions we show that, approximately (see proof below),

$$\begin{aligned} \Lambda(t) &= \kappa_n N C^{n-1} \nu_0^n \frac{1}{a_0 (n+1)!} (a_0 + t)^{n+1}, \quad -a_0 \leqslant t \leqslant 0, \\ \Lambda(t) &= \kappa_n N C^{n-1} \left(\nu_0^n \sum_{k=0}^{n-1} \frac{a_0^{n-k}}{k! \, (n+1-k)!} t^k + \nu^n \frac{1}{n!} t^n \right), \quad t \geqslant 0. \end{aligned} \tag{20}$$

Note that since $\nu_0 = (2 - p_0) u b_0$ and $\nu = (2-p) ub$ this implies in particular that for $t \geqslant 0$ the cumulative hazard is of the form

$$\Lambda(t) = N u^n \left(b_0^n \sum_{k=0}^{n-1} c_k t^k + b^n c_n t^n \right),$$

where the coefficients $c_0, \dots c_n$ do not depend on b_0, b, u, N.

Weibull distribution Let T_n denote the age at which cancer arises. According to our model, T_n is a random variable with values in $[-a_0, +\infty)$ and cumulative distribution function $P(t) = 1 - e^{-\Lambda(t)}$. We define T_n^+ as T_n conditioned on being positive, i.e. on the event $\{T_n \geqslant 0\}$ that cancer occurs after birth. Similarly, T_n^- is defined as T_n conditioned on the event $\{T_n \leqslant 0\}$ that cancer occurs between conception and birth.

We prove below that T_n is the minimum of independent Weibull random variables. For each $k = 0 \dots n$, let W_k denote the age at which cancer arises knowing that exactly $n-k$ out of the n required driver mutations occurred before birth. Note that by definition W_0 is negative and each W_k is positive for $k \geqslant 1$. Since cancer arises before time t if and only if at least one of these scenarios leads to cancer before t, we immediately have

$$\begin{cases} T_n^+ \sim \min(W_1, \dots, W_n), \\ T_n^- \sim W_0. \end{cases}$$

The notation $\sim$ means "follows the distribution". The fact that the W_k are Weibull random variables is an immediate consequence of (20). Indeed, denoting by $\mathcal{W}(\alpha, \beta)$ the Weibull distribution with cumulative distribution function $t \mapsto 1 - e^{-\beta t^\alpha}$, and defining

$$W_n \sim \mathcal{W}\left(n, \frac{\kappa_n N C^{n-1} \nu^n}{n!}\right),$$

$$W_k \sim \mathcal{W}\left(k, \frac{a_0^{n-k} \kappa_n N C^{n-1} \nu_0^n}{k! \, (n+1-k)!}\right), \quad 1 \leqslant k \leqslant n-1,$$

and

$$W_0 \sim \tilde{W}_0 - a_0 \mid \{\tilde{W}_0 \leqslant a_0\},$$

the translated and truncated Weibull distribution with values in $[-a_0, 0]$, where

$$\tilde{W}_0 \sim \mathcal{W}\left(n+1, \frac{\kappa_n N C^{n-1} \nu_0^n}{a_0 (n+1)!}\right),$$

we deduce from (20) that $\mathbb{P}(T_n \leqslant 0) = 1 - e^{-\kappa_n N C^{n-1} \nu_0^n a_0^n / (n+1)!}$, leading for each $t \geqslant 0$ to

$$\begin{aligned} \mathbb{P}\left(T_n^+ \leqslant t\right) &:= \mathbb{P}(T_n \leqslant t \mid T_n \geqslant 0) = 1 - \frac{\mathbb{P}(T_n > t)}{\mathbb{P}(T_n \geqslant 0)} \\ &= 1 - e^{-\kappa_n N C^{n-1} \left(\nu_0^n \sum_{k=1}^{n-1} a_0^{n-k} t^k / k! (n+1-k)! + \nu^n t^n / n! \right)} \\ &= 1 - \mathbb{P}(W_1 > t) \dots \mathbb{P}(W_n > t) \\ &= \mathbb{P}(\min(W_1, \dots, W_n) \leqslant t). \end{aligned}$$

Similarly, for each $-a_0 \leqslant t \leqslant 0$,

$$\mathbb{P}\left(T_n^- \leqslant t\right) = \frac{\mathbb{P}\left(T_n \leqslant t\right)}{\mathbb{P}\left(T_n \leqslant 0\right)} = \frac{1 - e^{-\kappa_n N C^{n-1} \nu_0^n (a_0+t)^{n+1}/a_0(n+1)!}}{1 - e^{-\kappa_n N C^{n-1} \nu_0^n a_0^n/(n+1)!}}$$
$$= \frac{\mathbb{P}\left(\tilde{W}_0 - a_0 \leqslant t\right)}{\mathbb{P}\left(\tilde{W}_0 \leqslant a_0\right)} = \mathbb{P}\left(W_0 \leqslant t\right).$$

Adult cancers In the particular case of cancer types for which the probability of driver mutations occurring before birth is negligible compared to the cancer risk, the cumulative hazard function (20) simplifies to

$$\Lambda\left(t\right) = \frac{1}{n!}\kappa_n N C^{n-1} \nu^n t^n, \tag{21}$$

hence with the proportionality relationship $\Lambda\left(t\right) \propto N C^{n-1} u^n b^n t^n$. The age T_n at which cancer arises is in this case therefore positive and follows a Weibull distribution

$$T_n \sim \mathcal{W}\left(n, \frac{\kappa_n N C^{n-1} \nu^n}{n!}\right). \tag{22}$$

Approximation (21) can be made if the cancer risk at birth $\rho_0 := \mathbb{P}\left(T_n \leqslant 0\right)$ is small enough that

$$-\ln\left(1 - \rho_0\right) \sum_{k=0}^{n-1} \binom{n+1}{k} \left(\frac{t}{a_0}\right)^k \ll \Lambda\left(t\right),$$

which can be easily verified for a known order of magnitude of the hazard function. Of course this approximation is not applicable to pediatric cancers.

Computation of the cumulative hazard function Let us prove (20). Under the aforementioned assumption of two distinct phases (before and after birth), the cumulative hazard (18) becomes for any $t \geqslant -a_0$,

$$\Lambda\left(t\right) = N \sum_{V \in \mathcal{M}} \pi_V \left(\nu_0 \beta_V^0\left(t\right) + \nu \beta_V\left(t\right)\right),$$

with

$$\beta_V^0\left(t\right) = \frac{1}{a_0} \int_{-a_0}^{\min(0,t)} \left(a_0 + s\right) P_V^0\left(t - s\right) ds, \quad \beta_V\left(t\right) = \int_0^{\max(0,t)} P_V\left(t - s\right) ds.$$

Here s corresponds to the appearance time of a first driver mutation V, which under our simplifying assumption of no initial growth phase of a nodule, corresponds also to the appearance time of a V-clone of size C_V. The quantities $P_V^0\left(t-s\right)$ and $P_V\left(t-s\right)$ are the probability for a V-clone to lead to cancer in less than $t-s$ time units, given that mutation V appears in the tissue population before or after birth, respectively (i.e. $-a_0 \leqslant s < 0$ or $s \geqslant 0$). Recall that in the first case, the growth and mutation parameters of the V-clone depend on p_0, b_0, while they depend on p, b in the second case.

By definition of $\mathcal{C}$, for any genotype G carrying n mutations, the probability of leading to cancer is immediately given by

$$P_G\left(t\right) = \begin{cases} 1, & G \in \mathcal{C}, \\ 0, & G \notin \mathcal{C}. \end{cases} \tag{23}$$

If a genotype G carries at most $n-1$ driver mutations, then under our assumptions the probability (17) becomes

$$P_G\left(t\right) = 1 - \exp\left(-\nu_G C_G \sum_{V \in \mathcal{M}} \pi_{GV} \int_0^t P_{GV}\left(t - s\right) ds\right) \tag{24}$$

In particular, (24) applied to a genotype carrying one single mutation V which appears before birth corresponds to P_V^0, keeping in mind that in this case $\nu_V C_V = \gamma_V \nu_0 C$.

For the sake of conciseness we prove (20) for $n \in \{1, 2, 3\}$ (which corresponds to the standard numbers of drivers in the studied cancers). The case $n = 1$ is an exact computation, while the case $n \in \{2, 3\}$ relies on Taylor's expansions. The methodology remains identical for any $n \geqslant 4$.

- $n=1$. By (23), for any $V\in\mathcal{M}$, $P_V=P_V^0=\mathbf{1}_{V\in\mathcal{C}}$ (the indicator function equal to 1 if $V\in\mathcal{C}$ and 0 otherwise), leading for $t\geqslant 0$ to $\beta_V(t)=\mathbf{1}_{V\in\mathcal{C}}t$, $\beta_V^0(t)=\frac{1}{2}a_0\mathbf{1}_{V\in\mathcal{C}}$ and consequently $\Lambda(t)=\kappa_1 N\left(\frac{1}{2}a_0\nu_0+\nu t\right)$ since $\kappa_1=\sum_{V\in\mathcal{C}}\pi_V$.

  Similarly if $-a_0\leqslant t\leqslant 0$, $\beta_V^0(t)=\frac{1}{2a_0}(a_0+t)^2\mathbf{1}_{V\in\mathcal{C}}$ and $\Lambda(t)=\kappa_1 N\nu_0\frac{1}{2a_0}(a_0+t)^2$.

- $n=2$. Combining (23) -(24) and the fact that $\nu_V C_V=\gamma_V\nu C$ we know that for any $V\in\mathcal{M}$, $P_V(t)=1-e^{-\alpha_V\nu Ct}$ and $P_V^0(t)=1-e^{-\alpha_V\nu_0 Ct}$, where

$$\alpha_V:=\gamma_V\sum_{U\in\mathcal{M}:\ VU\in\mathcal{C}}\pi_{VU}.$$

  Note that $\sum_{V\in\mathcal{M}}\alpha_V\pi_V=\kappa_2$. Then, for any $t\geqslant 0$

$$\beta_V(t)=\frac{e^{-\alpha_V\nu Ct}-1+\alpha_V\nu Ct}{\alpha_V\nu C}=\frac{1}{2}\alpha_V\nu Ct^2\left(1+\varepsilon_V(t)\right),$$

$$\begin{aligned}\beta_V^0(t)&=\frac{e^{-\alpha_V\nu_0 Ct}\left(1-\alpha_V\nu_0 Ca_0-e^{-\alpha_V\nu_0 Ca_0}\right)+\frac{1}{2}\left(\alpha_V\nu_0 Ca_0\right)^2}{a_0\left(\alpha_V\nu_0 C\right)^2}\\&=\frac{1}{6}\alpha_V\nu_0 Ca_0\left(a_0+3t\right)\left(1+\varepsilon_V^0(t)\right),\end{aligned}$$

  where $|\varepsilon_V(t)|$ and $\left|\varepsilon_V^0(t)\right|$ are negligible compared to 1 as soon as $\alpha_V\nu Ct\ll 1$, $\alpha_V\nu_0 C(a_0+t)\ll 1$. It ensues that

$$\Lambda(t)=\kappa_2 NC\left(\nu_0^2\frac{1}{6}a_0\left(a_0+3t\right)+\nu^2\frac{1}{2}t^2\right)+\varepsilon(t),$$

  where if for all $V\in\mathcal{M}$ $\alpha_V\nu Ct\ll 1$ and $\alpha_V\nu_0 C(a_0+t)\ll 1$, $|\varepsilon(t)|$ is negligible compared to the first right-hand term, leading to approximation (20). Since $a_0+t\leqslant 80$, this condition is satisfied in particular if $\gamma_V\nu C$ is at most of the order of 10^{-2} for all $V\in\mathcal{M}$.

  We similarly obtain that for any $-a_0\leqslant t\leqslant 0$,

$$\begin{aligned}\beta_V^0(t)&=\frac{1-\alpha_V\nu_0 C(a_0+t)+\frac{1}{2}\left(\alpha_V\nu_0 C(a_0+t)\right)^2-e^{-\alpha_V\nu_0 C(a_0+t)}}{a_0\left(\alpha_V\nu_0 C\right)^2}\\&=\frac{1}{3!a_0}\alpha_V\nu_0 C(a_0+t)^3\left(1+\varepsilon_V^0(t)\right),\end{aligned}$$

  hence $\Lambda(t)=\kappa_2 NC\nu_0^2\frac{1}{3!a_0}(a_0+t)^3+\varepsilon(t)$.

- $n=3$. We define for each genotype VU with two driver mutations the constant

$$\alpha_{VU}:=\gamma_V\gamma_U\sum_{W:\ VUW\in\mathcal{C}}\pi_{VUW}.$$

  Note in particular that $\sum_V\sum_U\gamma_V\pi_V\pi_{VU}\alpha_{VU}=\kappa_3$. We deduce from (23) -(24) that for each $V\in\mathcal{M}$ and $t\geqslant 0$,

$$\beta_V(t)=\int_0^t\left(1-e^{-\nu_V C_V\sum_{U\in\mathcal{M}}\pi_{VU}\int_0^{t-s}\left(1-e^{-\alpha_{VU}\nu C(t-s-z)}\right)dz}\right)ds.$$

  which has the form

$$\beta_V(t)=\int_0^t\left(1-e^{-\sum_U b_U\left(s-\frac{1}{a_U}\left(1-e^{-a_U s}\right)\right)}\right)ds,$$

  where $b_U=\gamma_V\pi_{VU}\nu C$, $a_U=\alpha_{VU}\nu C$. Computing the successive derivatives of β_V we obtain that $\beta_V(0)=\beta_V^{(1)}(0)=\beta_V^{(2)}(0)=0$, $\beta_V^{(3)}(0)=\sum_U a_U b_U$ and

$$\left|\beta_V^{(4)}(t)\right|\leqslant\sum_U a_U^2 b_U+3\sum_U a_U b_U\sum_U b_U+\left(\sum_U b_U\right)^3.$$

It follows from Taylor's theorem that $\beta_V(t) = \frac{1}{3!}\sum_U a_U b_U t^3 (1+\varepsilon_V(t))$ with

$$\begin{aligned}|\varepsilon_V(t)| &\leqslant \frac{1}{4}\frac{\sum_U a_U^2 b_U + 3\sum_U a_U b_U \sum_U b_U + \left(\sum_U b_U\right)^3}{\sum_U a_U b_U} t \\ &= \frac{\gamma_V \nu C}{4}\frac{\sum_U \left(\tilde{a}_U^2 + 3\tilde{a}_U + 1\right)\pi_{VU}}{\sum_U \tilde{a}_U \pi_{VU}} t,\end{aligned}$$

where $\tilde{a}_U = \gamma_U \sum_{W:\ VUW\in\mathcal{C}} \pi_{VUW}$. Given that the multiplicative effects of F and M mutations on νC are generally small compared to the effect of a S mutation (namely, in Table S1, $\gamma_S \gg \gamma_F, \gamma_M$), the right-hand side of the previous inequality is of the order of $\frac{\gamma_V \nu C}{4}(\gamma_S + 3)t$. In particular, if $\gamma_V \gamma_S \nu C \leqslant 10^{-2}$ then $|\varepsilon_V(t)| \ll 1$. Coming back to the original notation, we thus have proven that

$$\beta_V(t) = \frac{1}{3!}\nu^2 C^2 \sum_U \gamma_V \pi_{VU} \alpha_{VU} t^3 (1+\varepsilon_V(t)).$$

Following the same reasoning for the computation of $\beta_V^0(t)$, we obtain for $t \geqslant 0$

$$\Lambda(t) = \kappa_3 N C^2 \left(\nu_0^3 \frac{1}{4!} a_0 \left(a_0^2 + 4a_0 t + 6t^2\right) + \nu^3 \frac{1}{3!} t^3\right) + \varepsilon(t),$$

where $\varepsilon(t)$ can be neglected if, for instance, $\nu_G C_G$ is at most of the order of 10^{-2} for all genotypes with at most 2 driver mutations, leading to approximation (20).

Similarly, we obtain for $-a_0 \leqslant t \leqslant 0$, $\Lambda(t) = \kappa_3 N C^2 \nu_0^3 \frac{1}{4! a_0}(a_0 + t)^4 + \varepsilon(t)$.

Parameter ranges For the studied cancer types and any genotype G with at most $n-1$ driver mutations, we assume the following orders of magnitude:

- $C_G \in \left[10^2, 10^4\right]$ stem cells. Indeed, using the common estimate of about 10^9 cells in 1 cm^3, with a ratio of 1 : 1000 between stem cells and fully differentiated cells, it follows that a typical colorectal adenoma (which is usually containing 2 driver hits, e.g. APC and KRAS, and is detected at a size of the order of a few cubic millimeters) will have around 10^3 stem cells. This number may be higher (10^4) due to the effect of a F driver mutation on the ratio between stem cells and fully differentiated cells. We use these values across cancer types as the average size of pre-cancer tumoral masses is expected to be comparable.

- $b_0, b_G \in \left[10^{-2}, 10^2\right]$ divisions per year (Table 1).

- $u \in \left[10^{-10}, 10^{-7}\right]$ driver gene mutations per cell division. This is deduced from the following facts: *i)* the mutation rate per nucleotide base per cell division is estimated to be $10^{-10} - 10^{-9}$ (see [12] and references therein); *ii)* the exome is about $3 \cdot 10^7$ bases long, with a number of genes being approximately $2 \cdot 10^4 - 3 \cdot 10^4$, from which we deduce that the average coding length of a gene is 1000 bases, which would yield an average mutation rate per cell division of 10^{-6}; *iii)* we consider 10 to be a reasonable upper bound for the possible number of driver genes at each one of the n driver events required to get cancer. In order to calculate the driver mutation rate u we need to distinguish between oncogenes and tumor suppressors. For an oncogene, the number of bases ("hot spots") that are potential targets for a driver mutation is typically very few $(1-10)$, which when using 10 as the number of possible driver genes gives us a driver mutation rate of at most $10^{-9} \times 10^2 = 10^{-7}$ for oncogenes. For a tumor suppressor however, almost all of the 1000 bases are a potential target for a mutation hit, but in this case a mutation in the other allele is required; either as a point mutation which is a rare event or, more commonly, via loss of heterozygosity (LOH), whose estimates are of a rate of $10^{-5} - 10^{-2}$ ([13]). The driver mutation rate for tumor suppressors is therefore at most $10^{-9} \times 10^4 \times 10^{-2} = 10^{-7}$ mutations per cell division.

- if a genotype G contains a M mutation, $u_G \in \left[10^{-10}, 10^{-6}\right]$ driver gene mutations per cell division, because in the presence of a type M mutation, the driver mutation rate has been observed to be even up to 10 times higher, e.g., in Lynch syndrome patients.

Given (13) we thus obtain the very broad interval $\nu_G C_G \in \left[10^{-11}, 10^0\right]$, and similarly for $\nu_G^0 C_G$. Note however that this interval is based on numerous, conservative and independent bounds that are in no cancer type reached all at once. Given the previous estimates it seems reasonable that in many

cases, $\nu_G C_G$ is of the order of 10^{-2} or less, implying that the remainders in Taylor's theorem can be neglected and that approximation (20) holds. For example, for colorectal cancer, the value of $\nu_G C_G$ would be about $2 \cdot 10^{-7} \times 10^2 \times 10^3 = 2 \cdot 10^{-2}$, unless the ratio of stem cells to fully differentiated cells changes significantly. Of course this implies that for Lynch syndrom patients, the remainder in the Taylor's expansion may possibly not be negligible. However, colorectal cancer represents an extreme scenario given its very high normal division rate. Essentially all other tissues have smaller division rates (see Table 1), going from 10 times smaller in blood, to 100 times smaller in pancreas, to a 1000 times smaller in lung and bone.

S4 A re-analysis of the role of endogenous mutations in cancer risk

In what follows we denote by T the life expectancy and $P = P(T)$ the cancer lifetime risk. Considering the most common cancer (breast cancer) and its life time risk 12.9%, it appears that for any cancer type $|P(T) - \Lambda(T))| \ll P(T)$. We thus use here the approximation $P = \Lambda(T)$ where Λ is given by (21) (all cancer types studied in this paper have an infinitesimal risk at birth). Letting $D = bT$ be the lifetime number of divisions we thus obtain $P = \frac{1}{n!}\kappa_n N C^{n-1} (2-p)^n u^n D^n$, and therefore the proportionality relationship

$$P \propto N D^n.$$

Note that if we assume that C is proportional to the full tissue size N (which may be the case for some small tissues) rather than being a local constraint, the relationship becomes $P \propto (ND)^n$. From these expressions, it is clear that in [16] the carrying capacity was treated as a global constraint. In general, however, the carrying capacity is acting more at the local level.

S5 Predicted number of mutations

The expected number of somatic mutations found in a cancer cell lineage of a patient of age a is

$$\tilde{\eta} = \mu\left(D_0 + bT_1 + b^{(1)}(T_2 - T_1) + \ldots + b^{(n)}(a - T_n)\right),$$

where D_0 is the number of cell divisions until birth, μ is the somatic mutation probability per cell division, and b_0, b, $b^{(i)}$, the cell division rates before birth, after birth, and after i driver hits. Recall that according to our classification of the driver gene mutations (Section S2.4), only S mutations increase the cell division rate. Therefore, if s is the fitness advantage of a S mutation (i.e. such that $\gamma_S = (1+s)^2$ in Table S1) and if k out of the i driver hits are of type S, then $b^{(i)} = (1+s)^k b$. While the above formula $\tilde{\eta}$ can be applied to single cell sequencing data, in the case of bulk sequencing we only observed the mutations accumulated in the winning lineage of the first cancer cell, born at time T_n, as all the subsequent mutations will not be called by the sequencer. In that case the appropriate formula is:

$$\eta = \mu\left(D_0 + bT_1 + b^{(1)}(T_2 - T_1) + \ldots + b^{(n-1)}(T_n - T_{n-1})\right). \tag{25}$$

The time between T_n and a is relatively small, being typically more than one order of magnitude smaller than the value of a. For example, while a typical value for a is 65-70 years, the difference $a - T_n$ is often estimated to be less than 2-5 years. Let c be an upper bound estimate for the amount of time it takes for the first cancer cell to get to detection size in that particular cancer type. In order to compare (25) with the observed number of mutations $\eta^{obs}(a)$ from sequencing data, we thus condition η on the event $\{a - c \leqslant T_n \leqslant a\}$ and compute

$$\eta^{exp}(a) = \mathbb{E}(\eta \mid a - c \leqslant T_n \leqslant a).$$

By (22), for each $t \geqslant 0$,

$$\begin{cases} \mathbb{P}(T_n \leqslant t) = 1 - e^{-\beta t^n}, \\ \beta = \frac{1}{n!}\kappa N C^{n-1} \nu^n. \end{cases} \tag{26}$$

It ensues $\mathbb{P}(a - c \leqslant T_n \leqslant a) = e^{-\beta(a-c)^n} - e^{-\beta a^n}$ and

$$\mathbb{E}\left(T_n \mathbf{1}_{a-c\leqslant T_n \leqslant a}\right) = \beta n \int_{a-c}^{a} t^n e^{-\beta t^n} dt = \beta^{-\frac{1}{n}} \int_{\beta(a-c)^n}^{\beta a^n} z^{\frac{1}{n}} e^{-z} dz,$$

which using the notation $\gamma(z;a,b) := \int_a^b t^{z-1}e^{-t}dt$ leads to

$$\mathbb{E}\left(T_n \mid a-c \leqslant T_n \leqslant a\right) = \beta^{-\frac{1}{n}} \frac{\gamma\left(1+\frac{1}{n}; \beta(a-c)^n, \beta a^n\right)}{\gamma\left(1; \beta(a-c)^n, \beta a^n\right)}. \tag{27}$$

For lack of better information on the joint distribution of $(T_1, \ldots, T_n)$ and since our goal is only to have a rough estimation of $\eta^{exp}(a)$, we assume that $T_i = \frac{i}{n}T_n$, leading to

$$\eta^{exp}(a) = \mu\left(D_0 + \frac{1}{n}\left(b + b^{(1)} + \ldots + b^{(n-1)}\right)\mathbb{E}\left(T_n \mid a-c \leqslant T_n \leqslant a\right)\right). \tag{28}$$

If $n = 1$, the formula is simply $\eta^{exp}(a) = \mu\left(D_0 + b\mathbb{E}\left(T_1 \mid a-c \leqslant T_1 \leqslant a\right)\right)$. Assume $n > 1$. Note that $b^{(n)} = (1+s)^k b$ where k is the (random) number of S mutations among the n drivers. Since $k \in \{1,2\}$ for $n \in \{2,3,4\}$, we assume that we equal probability $\frac{2}{n(n+1)}$ a S mutation occurs only at one given hitting time T_i, $i = 1, \ldots, n$, or at two hitting times $\{T_i, T_j\}$, $i \neq j$. We then obtain by averaging uniformly over the different occurrences of S mutations that

$$b^{(n)} = \left(\frac{2}{n+1}(1+s) + \frac{n-1}{n+1}(1+s)^2\right)b,$$

leading to the following average fitness of a S mutation

$$s = \frac{1}{n-1}\left(\sqrt{1 + (n^2-1)\frac{b^{(n)}}{b}} - n\right).$$

The mutation rates $b^{(i)}$ for $i < n$ are obtained similarly by averaging over the S mutations occurrences, leading to

$$n = 2: \quad \begin{cases} b^{(1)} = \left(\frac{1}{3} + \frac{2}{3}(1+s)\right)b, \end{cases}$$

$$n = 3: \quad \begin{cases} b^{(1)} = \left(\frac{1}{2} + \frac{1}{2}(1+s)\right)b, \\ b^{(2)} = \left(\frac{1}{6} + \frac{2}{3}(1+s) + \frac{1}{6}(1+s)^2\right)b, \end{cases}$$

$$n = 4: \quad \begin{cases} b^{(1)} = \left(\frac{3}{5} + \frac{2}{5}(1+s)\right)b, \\ b^{(2)} = \left(\frac{3}{10} + \frac{3}{5}(1+s) + \frac{1}{10}(1+s)^2\right)b, \\ b^{(3)} = \left(\frac{1}{10} + \frac{3}{5}(1+s) + \frac{3}{10}(1+s)^2\right)b. \end{cases}$$

Remark. The quantity $\eta^{exp}(a)$ given by (28) is approximately linear in a. Indeed, since the lifetime cancer risk is small, it ensues that $\beta T^n \ll 1$ hence $\beta a^n \ll 1$. Using $\gamma(z; 0, t) \overset{t\to 0}{\sim} t^z/z$ enables us to approximate (27) with

$$\mathbb{E}\left(T_n \mid a-c \leqslant T_n \leqslant a\right) \approx \frac{n}{n+1}\frac{a^{n+1} - (a-c)^{n+1}}{a^n - (a-c)^n},$$

which does not depend on β. If in addition c is small compared to a, we can further use the linear approximation $\mathbb{E}\left(T_n \mid a-c \leqslant T_n \leqslant a\right) \approx a - \frac{c}{2}$ leading to

$$\eta^{exp}(a) = \mu\left(D_0 + \frac{1}{n}\left(b + b^{(1)} + \ldots + b^{(n-1)}\right)\left(a - \frac{c}{2}\right)\right). \tag{29}$$

Comparison with sequencing data. Given n, D_0, b and $b^{(n)}$ in a cancer tissue, ρ the proportion of the population that gets cancer by age 80 (see Table 1) obtained from epidemiological data (SEER Cancer Statistics Review, 1975-2018, https://seer.cancer.gov/csr/1975_2018/index.html), we can explicitly compute (28). Indeed, it comes from (26) that $\beta = -\ln(1-\rho)/80^n$. This enables us to compare the observed number of somatic mutations found in a cancer patient of age a obtained from the TCGA database (The Cancer Genome Atlas, https://www.cancer.gov/tcga) and the ICGC (International Cancer Genome Consortium, https://dcc.icgc.org) with the expected number in the absence of E and H factors for the same age and cancer, given by (28). This comparison is depicted for the TCGA observations in Fig. 1b ($n = 3$) and in Fig. S3 for each single cancer type ($n = 2, 3, 4$). In each case, the values for c in (28) is 5 years, and the background mutation rate is $\mu = 0.03$ somatic mutations per cell division. In addition, in order to illustrate the impact of some known environmental factors we include the effect of tobacco smoking and UV light by assuming that the actual mutation rate in lungs and skin is 4μ. The comparison between the observed and expected number of mutations under this assumption is plotted for the TCGA and ICGC databases in Fig. 1c-1d ($n = 3$) and Fig. S2 ($n = 2$).

(a)

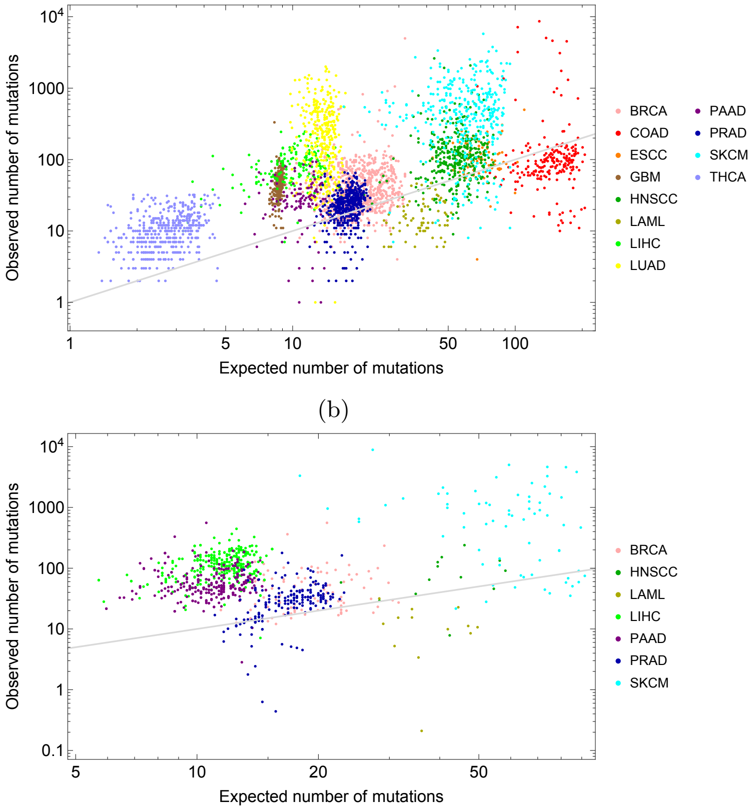

(b)

Figure S2: **Predicted versus observed number of mutations.** Observed number of somatic mutations found in: **(a)** 3608 cancers, for 12 types of cancer (TCGA database), **(b)** 990 cancers, for 7 types of cancer (ICGC database), versus the corresponding expected number of mutations solely due to the endogenous mutational processes as predicted by our model for a cancer patient of the same age (see Table 2 for the meaning of the abbreviations). For lung cancer (LUAD) and melanoma (SKCM), we include the effect of tobacco smoking and UV light, respectively. The number of drivers for each cancer type is assumed to be $n = 2$ (see Fig. 1c-1d for $n = 3$). The grey identity line corresponds to the theoretical case "observed number of mutations" = "expected number of mutations". Source data are provided as a Source Data file.

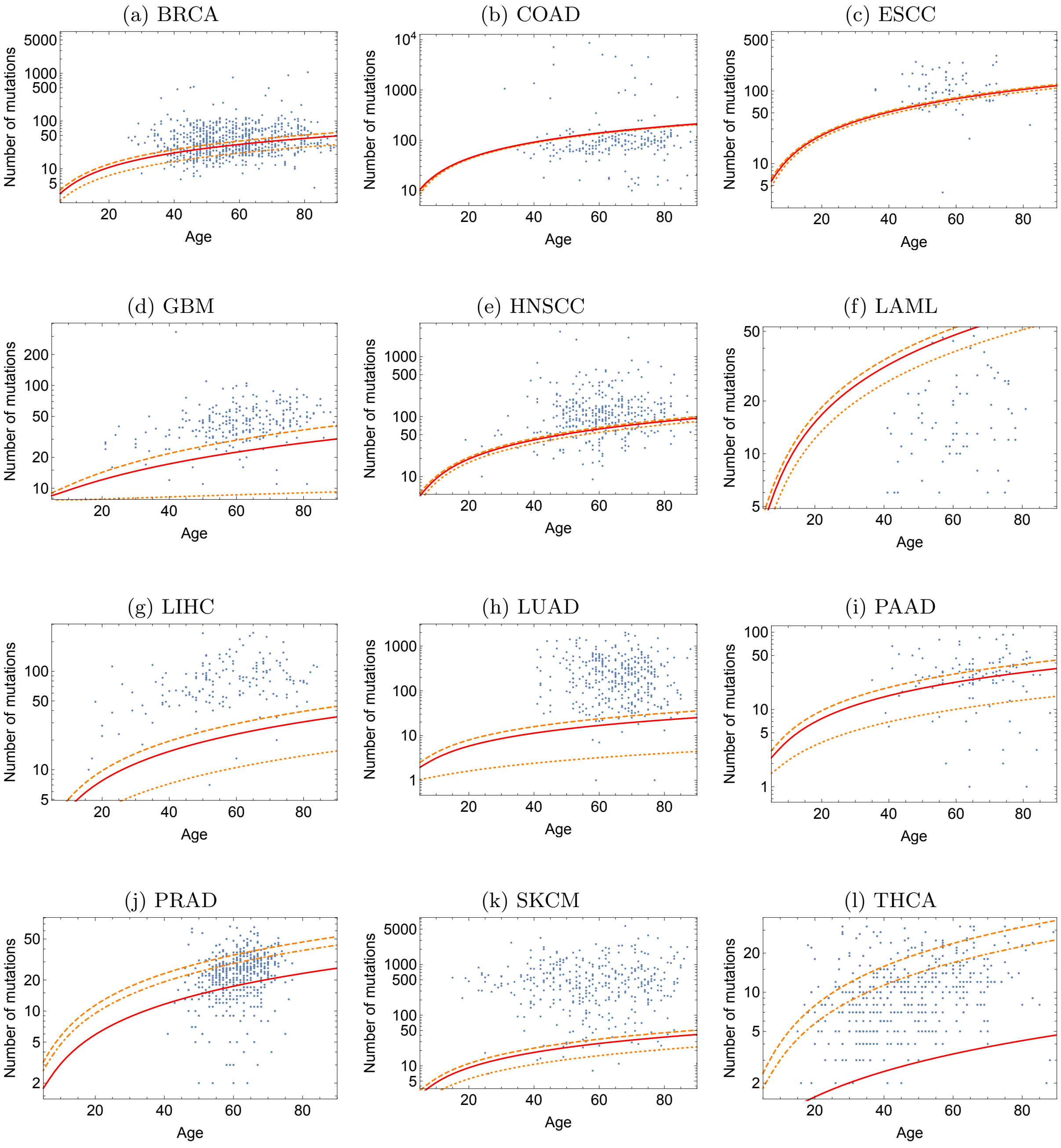

Figure S3: **Predicted versus observed number of mutations as a function of age.** Observed number of somatic mutations found in cancers (blue dots) of patients of different ages from the TCGA database, compared with the expected number of mutations solely due to the endogenous mutational processes in patients of the same age, predicted by our model (orange and red lines), for 12 types of cancer (see Table 2 for the meaning of the abbreviations). The expected number of mutations is given for a number of drivers equal $n = 2$ (dotted orange line), $n = 3$ (dash-dotted orange line) and $n = 4$ (dashed orange line). The solid red line corresponds to the computation for the value of n deduced from [15] and given in Section S6. Source data are provided as a Source Data file.

Measure of the deviation of the predictions from the observations. For a given cancer type and a given set of patients with this cancer, we measure the difference between the observations $\{\eta_i^{obs}(a_i)\}_i$ (where $\eta_i^{obs}(a_i)$ is the observed number of mutations for patient i aged a_i) and the corresponding expected number of mutations $\{\eta^{exp}(a_i)\}_i$ given by (28) for some fixed n. The deviation δ is evaluated as the relative difference between the medians of the set of observed and expected number of mutations weighted by the patient's age:

$$\delta = \frac{\left|\text{Median}\left(\{\eta^{exp}(a_i)/a_i\}_i\right) - \text{Median}\left(\{\eta_i^{obs}(a_i)/a_i{}_i\right)\right|}{\text{Median}\left(\{\eta_i^{obs}(a_i)/a_i{}_i\right)}.$$

The deviation δ for each cancer type and for different databases (TCGA, ICGC) is reported in Table S2. Note in particular the high deviation for lung cancer (LUAD) and melanoma (SKCM), in accordance with the fact that these cancer types are clear outliers in Fig. 1b. If environmental risk factors are taken into account by increasing the mutation rate in the corresponding tissues, the deviation is diminished, as also observed in Fig. 1c.

	TCGA				ICGC			
Cancer name	$n=1$	$n=2$	$n=3$	$n=4$	$n=1$	$n=2$	$n=3$	$n=4$
BRCA	0.78	0.45	0.17	**0.02**	0.82	0.55	0.32	**0.20**
COAD	**0.51**	0.61	0.65	0.66	-	-	-	-
ESCC	0.45	0.31	0.26	**0.23**	-	-	-	-
GBM	0.83	0.81	0.50	**0.34**	-	-	-	-
HNSCC	0.63	0.46	0.39	**0.35**	0.59	0.42	0.33	**0.29**
LAML	**0.46**	1.58	2.20	2.53	**1.37**	3.18	4.20	4.73
LIHC	0.97	0.87	0.72	**0.65**	0.97	0.90	0.77	**0.71**
LUAD	0.99	0.98	0.91	**0.87**	-	-	-	-
LUAD*	0.98	0.93	0.64	**0.49**	-	-	-	-
PAAD	0.90	0.61	**0.12**	0.13	0.95	0.79	0.52	**0.39**
PRAD	0.74	0.25	**0.25**	0.50	0.80	0.41	**0.02**	0.18
SKCM	0.99	0.97	0.94	**0.93**	0.99	0.98	0.96	**0.96**
SKCM*	0.96	0.87	0.77	**0.72**	0.97	0.92	0.86	**0.83**
THCA	0.91	0.74	**0.26**	0.76	-	-	-	-

Table S2: **Deviation of the predictions from the observations**. Measure of the deviation δ of the expected number of mutations from the observed number of mutations for different cancer types of the TCGA and ICGC databases, for a fixed number of drivers n (see Table 2 for the meaning of the abbreviations). The symbol * indicates that the expected number of mutations has been computed by using a higher mutation rate (a four-fold increase of the background mutation rate) to account for the effect of known environmental risk factors such as tobacco smoking (LUAD) and UV light (SKCM). The lowest deviation δ for a given cancer type is shown in bold. The resulting optimal choice for n is not reliable in cases in which the changes in the deviation are small when varying n (e.g. COAD).

Optimal background mutation rate. Computing the average of the deviation δ over all cancer types, we obtain that for $n = 3$ this mean deviation is minimized if the background mutation rate is equal to 0.024 (Fig. 2e). We obtain the same result if the average is taken over all cancer types except for the outliers LUAD and SKCM. This is striking as this estimate is very close to the parameter value $\mu = 0.03$ we originally chose for our model as provided by the available literature.

S6 Multidimensional 3D analysis

S6.1 Original analysis from Tomasetti and Vogelstein (Science, 2015)

From the 31 datapoints used in [16] Figure 1, we subtract six environmental or inherited subgroup cancer types (as explained in Tomasetti et al. Technical Report 2015), because they are just subgroups of cancer types already included in the analysis. Specifically, we remove FAP colorectal, FAP duodenum, HCV hepatocellular, HPV-16 head and neck, Lynch colorectal, and combine lung cancers of smokers and nonsmokers together. We also do not use the overall lifetime risk of lung adenocarcinoma (0.069) but rather the one for never-smokers (0.0045) in order not to bias the analysis with a strong environmental factor. Moreover, we keep legs, arms, head, and pelvis osteosarcomas, as they are cancers from separate locations, but remove osteorsacomas to eliminate any overlap, resulting in a total of 24 cancer types. Finally, we add breast and prostate cancers, as estimated in Tomasetti et al. Science 2017 [17], yielding a total of 26 different cancer types.

We list here the name, lifetime risk (estimated at age 80), and total number of cell divisions for each of those 26 cancer types.

```
names <- c("Acute myeloid leukemia", "Arms osteosarcoma", "Basal cell carcinoma",
    "Breast Cancer", "Chronic lymphocytic leukemia", "Colorectal adenocarcinoma",
    "Duodenum adenocarcinoma", "Esophageal squamous cell carcinoma",
    "Gallbladder adenocarcinoma", "Glioblastoma", "Head_Neck squamous cell carcinoma",
    "Head osteosarcoma", "Hepatocellular carcinoma", "Legs osteosarcoma",
    "Lung adenocarcinoma", "Medulloblastoma", "Melanoma", "Ovarian germ cell",
    "Pancreatic ductal adenocarcinoma", "Pancreatic endocrine carcinoma",
    "Pelvis osteosarcoma", "Prostate Cancer", "Small intestine adenocarcinoma",
    "Testicular germ cell", "Thyroid follicular_papillary", "Thyroid medullary")

liferisk <- c(0.0041, 4e-05, 0.3, 0.129, 0.0052, 0.048, 3e-04,
    0.001938, 0.0028, 0.00219, 0.0138, 3.02e-05, 0.0071, 0.00022,
    0.0045, 0.00011, 0.0203, 0.000411, 0.013589, 0.000194, 3e-05,
    0.121, 7e-04, 0.0037, 0.01026, 0.000324)
```

```
cellsdiv <- c(1.35 * 10^8 * 960 + sum(2^(1:floor(log2(1.35 *
    10^8)))), 6.5 * 10^5 * 5 + sum(2^(1:floor(log2(6.5 * 10^5)))),
    5.82 * 10^9 * 608 + sum(2^(1:floor(log2(5.82 * 10^9)))),
    0.016 * 0.8 * 6.8 * 10^11 * 346 + sum(2^(1:floor(log2(0.016 *
        0.8 * 6.8 * 10^11)))), 1.35 * 10^8 * 960 + sum(2^(1:floor(log2(1.35 *
        10^8)))), 2 * 10^8 * 5840 + sum(2^(1:floor(log2(2 * 10^8)))),
    4 * 10^6 * 1947 + sum(2^(1:floor(log2(4 * 10^6)))), (6.7 *
        10^6) * 2655 + sum(2^(1:floor(log2(6.7 * 10^6)))), 1.6 *
        10^6 * 47 + sum(2^(1:floor(log2(1.6 * 10^6)))), 1.35 *
        10^8 * 0 + sum(2^(1:floor(log2(1.35 * 10^8)))), 1.85 *
        10^7 * 1720 + sum(2^(1:floor(log2(1.85 * 10^7)))), 8.6 *
        10^5 * 5 + sum(2^(1:floor(log2(8.6 * 10^5)))), 3.01 *
        10^9 * 88 + sum(2^(1:floor(log2(3.01 * 10^9)))), 1.59 *
        10^6 * 5 + sum(2^(1:floor(log2(1.59 * 10^6)))), 1.22 *
        10^9 * 5.6 + sum(2^(1:floor(log2(1.22 * 10^9)))), 1.36 *
        10^8 * 0 + sum(2^(1:floor(log2(1.36 * 10^8)))), 3.8 *
        10^9 * 199 + sum(2^(1:floor(log2(3.8 * 10^9)))), 1.1 *
        10^7 * 0 + sum(2^(1:floor(log2(1.1 * 10^7)))), 4.18 *
        10^9 * 80 + sum(2^(1:floor(log2(4.18 * 10^9)))), 7.4 *
        10^7 * 80 + sum(2^(1:floor(log2(7.4 * 10^7)))), 4.5 *
        10^5 * 5 + sum(2^(1:floor(log2(4.5 * 10^5)))), 0.007 *
        30 * 10^9 * 240 + sum(2^(1:floor(log2(0.007 * 30 * 10^9)))),
    10^8 * 2920 + sum(2^(1:floor(log2(10^8)))), 7.2 * 10^6 *
        463 + sum(2^(1:floor(log2(7.2 * 10^6)))), 6.5 * 10^7 *
```

```
    7 + sum(2^(1:floor(log2(6.5 * 10^7)))), 6.5 * 10^6 *
    7 + sum(2^(1:floor(log2(6.5 * 10^6)))))
```

The numbers above result in the following correlation:

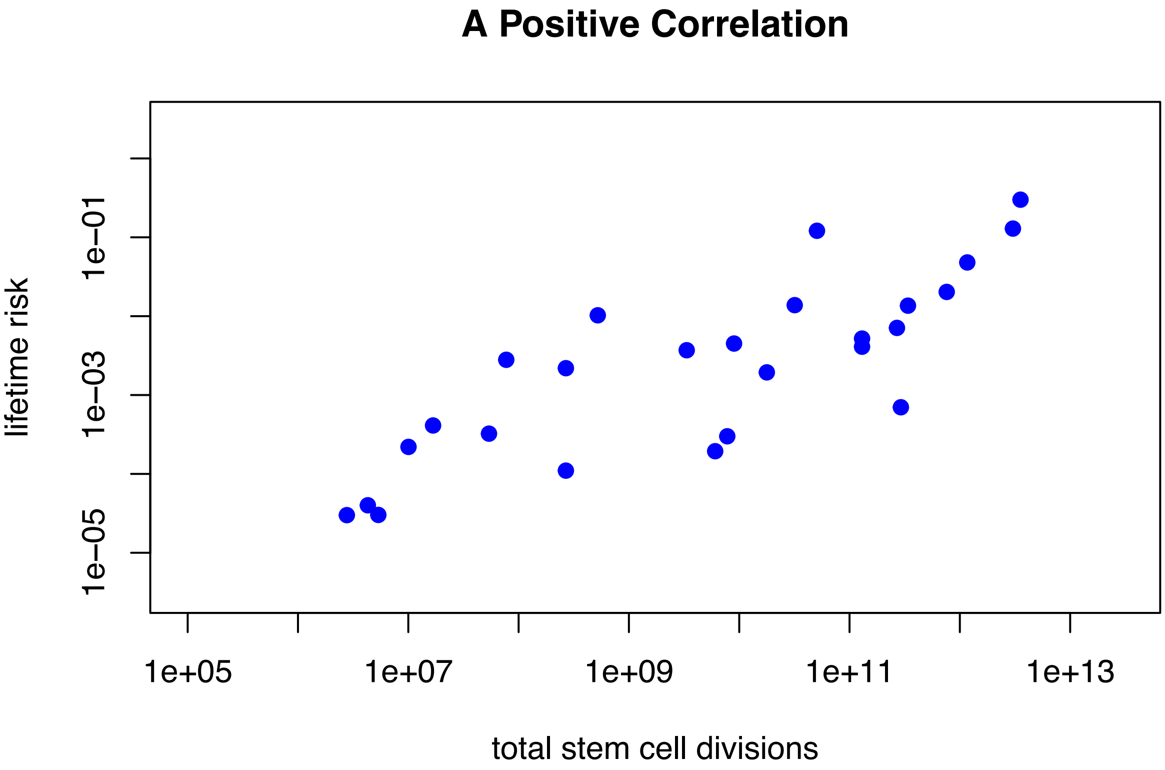


```
## Warning in cor.test.default(cellsdiv, liferisk, method = "spearman", exact =
## NULL): Cannot compute exact p-value with ties

##
##  Spearman's rank correlation rho
##
## data:  cellsdiv and liferisk
## S = 509.17, p-value = 2.04e-07
## alternative hypothesis: true rho is not equal to 0
## sample estimates:
##       rho
## 0.8259234
```

and running a linear regression yields the following results:

```
##
## Call:
## lm(formula = log10(liferisk) ~ log10(cellsdiv))
##
## Residuals:
##      Min       1Q   Median       3Q      Max
## -1.34533 -0.35328 -0.00388  0.40810  1.24888
##
## Coefficients:
##                 Estimate Std. Error t value Pr(>|t|)
## (Intercept)     -7.18227    0.67856 -10.585 1.60e-10 ***
```

```
## log10(cellsdiv)  0.46859    0.06882   6.809 4.83e-07 ***
## ---
## Signif. codes:  0 '***' 0.001 '**' 0.01 '*' 0.05 '.' 0.1 ' ' 1
##
## Residual standard error: 0.672 on 24 degrees of freedom
## Multiple R-squared:  0.6589, Adjusted R-squared:  0.6447
## F-statistic: 46.36 on 1 and 24 DF,  p-value: 4.834e-07
```

An important observation is that the slope of those points is much lower than 1, specifically the slope is 0.47. Theoretically we would expect a slope higher than, or equal to, 1.

S6.2 Multidimensional analysis

We now consider separately the total number of stem cells in a tissue and the total number of divisions in the lifetime of that tissue.

```
cells <- c(1.35 * 10^8, 6.5 * 10^5, 5.82 * 10^9, 0.016 * 0.8 *
    6.8 * 10^11, 1.35 * 10^8, 2 * 10^8, 4 * 10^6, 6.7 * 10^6,
    1.6 * 10^6, 1.35 * 10^8, 1.85 * 10^7, 8.6 * 10^5, 3.01 *
        10^9, 1.59 * 10^6, 1.22 * 10^9, 1.36 * 10^8, 3.8 * 10^9,
    1.1 * 10^7, 4.18 * 10^9, 7.4 * 10^7, 4.5 * 10^5, 0.007 *
        30 * 10^9, 10^8, 7.2 * 10^6, 6.5 * 10^7, 6.5 * 10^6)

div <- as.integer(c(960, 5, 608, 346, 960, 5840, 1947, 2655,
    47, 0, 1720, 5, 88, 5, 5.6, 0, 199, 0, 80, 80, 5, 240, 2920,
    463, 7, 7))
```

As always, we must add to the vector "div" above also the number of divisions that occur during development:

```
divwithdev <- div + floor(log2(cells))

divwithdev
```

```
##  [1]  987   24  640  379  987 5867 1968 2677   67   27 1744   24  119   25   35
## [16]   27  230   23  111  106   23  267 2946  485   32   29
```

We want to use multivariate regression now. To use the formula in the main text we need an estimate for u, the probability of a driver mutation per cell division, meaning the probability that during a cell division a driver mutation occurs. The mutation rate (per nucleotide base per cell division) has been estimated to be ~10^{-9}, and the average gene length is ~1000 bases as there are ~30000 genes in the exome and the length of the exome is $3 * 10^7$. This would yield a per driver gene mutation rate equal to $u = 10^{-6}$ in the case of a tumor suppressor (as all bases may be hit), and $u = 10^{-8}$ in the case of an oncogene with up to ten "hot spots". However, a tumor suppressor must be hit in both alleles, and given that the rate of loss of heterozygosity (LOH) is $< 10^{-2}$, we use $u = 10^{-8}$ for both tumor suppressors and oncogenes. As there are multiple drivers genes that can be hit, with the list of known driver genes being in the hundreds but only a subset of them found in any given cancer type, we assume for simplicity that the order of magnitude is 10, resulting in a final $u = 10^{-7}$. Note that the estimate for u will only affect the intercept, which is not the main focus of our analysis.

```
u = 10^(-7)
```

We also need to include the 3rd independent variable, n, as indicated by the formula, i.e.the number of required driver hits. The previous regression assumed that all cancer types listed have the same number of required driver hits, which is not correct as it is known that that number varies across tissues. While no established methodology is available today for determining the number of required drivers, n, typical in a given cancer type, there are a few papers attempting to estimate that number. Of them, possibly the one that looked at the largest number of cancer types is provided by the analysis produced by the Koonin's group (see Figure 4b in Iranzo PNAS 2018 [15]), and so we decided to use those results, both extending them

as explained below, as well as extrapolating from them to other tissues when no information for them was provided. Specifically, in that paper, it is reported that leukemia is estimated to have 2 driver mutations on average, sarcoma (like osteosarcoma) has 1, basal is NA so we set=2 as basal is very common so it should not have more drivers than melanoma, which has 2, breast 2, colon 3, duodenum is NA but it is next to and similar to the colon, so we set it equal to 3 as colon, esophageal 2, gallbladder NA but in another study the average is just a bit more than 1, glioblastoma 2, head and neck 2, hepatocellular 2, lung adenocarcinoma 2, medulloblastoma NA so we set it equal to 2, as glioblastoma is 2 and medulloblastoma should not have more drivers than glioblastoma (given the division rate), melanoma 2, ovarian germ cell is NA but ovary has a value just above 1 and testicular is 1 so we set it equal to 1, pancreas is 2-3 (more 2 than 3) so we assume 2 for both, prostate 1, small intestine NA so 3 as colon, testicular 1, thyroid 1.

This gives us the following vector of values:

```
drivers <- c(2, 1, 2, 2, 2, 3, 3, 2, 1, 2, 2, 1, 2, 1, 2, 2,
    2, 1, 2, 2, 1, 1, 3, 1, 1, 1)
```

While the approach in Iranzo et al. provides us with a range of values for n, across different tissues, and that is desirable and in fact even needed for our analysis, a major concern is that overall that analysis assigned a very low n to many tissues, with about half of the cancer types considered in Figure 4b of Iranzo et al. are estimated to have only one driver (n=1), which we think is too low. Therefore we increase all of those estimates for n by 1, purposely in a unbiased way, in order to keep the needed range. The resulting vector for n is then given by:

```
drivers <- drivers + 1
```

We now put all the data in a dataframe, adding a short code name to each cancer type.

```
codename <- c("LAML", "AOS", "BAS", "BRCA", "CLL", "COAD", "DUO",
    "ESCC", "GALL", "GBM", "HNSCC", "HOS", "LIHC", "LOS", "LUAD",
    "MEDB", "SKCM", "OVG", "PAAD", "PAE", "POS", "PRAD", "SMAD",
    "TES", "THF", "THM")


badluckdata <- data.frame(names, liferisk, cells, divwithdev,
    drivers, codename)

badluckdata
```

```
##                                   names  liferisk     cells divwithdev drivers
## 1               Acute myeloid leukemia 0.0041000 1.350e+08        987       3
## 2                   Arms osteosarcoma 0.0000400 6.500e+05         24       2
## 3                Basal cell carcinoma 0.3000000 5.820e+09        640       3
## 4                       Breast Cancer 0.1290000 8.704e+09        379       3
## 5         Chronic lymphocytic leukemia 0.0052000 1.350e+08        987       3
## 6             Colorectal adenocarcinoma 0.0480000 2.000e+08       5867       4
## 7              Duodenum adenocarcinoma 0.0003000 4.000e+06       1968       4
## 8  Esophageal squamous cell carcinoma 0.0019380 6.700e+06       2677       3
## 9           Gallbladder adenocarcinoma 0.0028000 1.600e+06         67       2
## 10                        Glioblastoma 0.0021900 1.350e+08         27       3
## 11  Head_Neck squamous cell carcinoma 0.0138000 1.850e+07       1744       3
## 12                   Head osteosarcoma 0.0000302 8.600e+05         24       2
## 13            Hepatocellular carcinoma 0.0071000 3.010e+09        119       3
## 14                   Legs osteosarcoma 0.0002200 1.590e+06         25       2
## 15                 Lung adenocarcinoma 0.0045000 1.220e+09         35       3
## 16                     Medulloblastoma 0.0001100 1.360e+08         27       3
## 17                            Melanoma 0.0203000 3.800e+09        230       3
## 18                   Ovarian germ cell 0.0004110 1.100e+07         23       2
```

```
## 19   Pancreatic ductal adenocarcinoma 0.0135890 4.180e+09        111       3
## 20     Pancreatic endocrine carcinoma 0.0001940 7.400e+07        106       3
## 21                Pelvis osteosarcoma 0.0000300 4.500e+05         23       2
## 22                    Prostate Cancer 0.1210000 2.100e+08        267       2
## 23     Small intestine adenocarcinoma 0.0007000 1.000e+08       2946       4
## 24               Testicular germ cell 0.0037000 7.200e+06        485       2
## 25       Thyroid follicular_papillary 0.0102600 6.500e+07         32       2
## 26                  Thyroid medullary 0.0003240 6.500e+06         29       2
##    codename
## 1      LAML
## 2       AOS
## 3       BAS
## 4      BRCA
## 5       CLL
## 6      COAD
## 7       DUO
## 8      ESCC
## 9      GALL
## 10      GBM
## 11    HNSCC
## 12      HOS
## 13     LIHC
## 14      LOS
## 15     LUAD
## 16     MEDB
## 17     SKCM
## 18      OVG
## 19     PAAD
## 20      PAE
## 21      POS
## 22     PRAD
## 23     SMAD
## 24      TES
## 25      THF
## 26      THM
```

Running the regression:

```
linereg = lm(log10(liferisk) ~ log10(cells) + log10((u * divwithdev)^drivers))
summary(linereg)
```

```
##
## Call:
## lm(formula = log10(liferisk) ~ log10(cells) + log10((u * divwithdev)^drivers))
##
## Residuals:
##     Min      1Q  Median      3Q     Max
## -0.6244 -0.3844 -0.1410  0.2200  0.9170
##
## Coefficients:
##                                 Estimate Std. Error t value Pr(>|t|)
## (Intercept)                     -6.03594    0.68532  -8.807 7.93e-09 ***
## log10(cells)                     0.90698    0.09093   9.975 8.01e-10 ***
## log10((u * divwithdev)^drivers)  0.28846    0.05297   5.446 1.55e-05 ***
## ---
```

```
## Signif. codes:  0 '***' 0.001 '**' 0.01 '*' 0.05 '.' 0.1 ' ' 1
##
## Residual standard error: 0.509 on 23 degrees of freedom
## Multiple R-squared:  0.8125, Adjusted R-squared:  0.7962
## F-statistic: 49.82 on 2 and 23 DF,  p-value: 4.371e-09
```

```
linereg = lm(log10(liferisk) ~ log10(cells) + log10(u^drivers) +
    log10(divwithdev^drivers))
summary(linereg)
```

```
##
## Call:
## lm(formula = log10(liferisk) ~ log10(cells) + log10(u^drivers) +
##     log10(divwithdev^drivers))
##
## Residuals:
##     Min      1Q  Median      3Q     Max
## -0.7344 -0.2955 -0.1363  0.3308  1.0003
##
## Coefficients:
##                           Estimate Std. Error t value Pr(>|t|)
## (Intercept)               -5.99405    0.62959  -9.521 2.93e-09 ***
## log10(cells)               0.86083    0.08588  10.024 1.16e-09 ***
## log10(u^drivers)           0.29504    0.04872   6.055 4.28e-06 ***
## log10(divwithdev^drivers)  0.35656    0.05696   6.259 2.67e-06 ***
## ---
## Signif. codes:  0 '***' 0.001 '**' 0.01 '*' 0.05 '.' 0.1 ' ' 1
##
## Residual standard error: 0.4674 on 22 degrees of freedom
## Multiple R-squared:  0.8487, Adjusted R-squared:  0.8281
## F-statistic: 41.15 on 3 and 22 DF,  p-value: 3.407e-09
```

S6.3 3D plot

```
library(scatterplot3d)

s3d = scatterplot3d(
  x = log10(badluckdata[,3]),
  y = log10((u*badluckdata[,4])^drivers),
  z = log10(badluckdata[,2]),
  pch=19,
  color="orange",
  angle = 55,
  # scale.x = 5.5,
  # scale.y = 5.5,
  # scale.z = 5.5,
  type = "h",
  xlab = "Log10(stem cells)",
  ylab = "Log10((u*total divisions)^drivers)",
  zlab = "Log10(lifetime risk)")

text(s3d$xyz.convert(
  x = log10(badluckdata[,3]),
  y = log10((u*badluckdata[,4])^drivers),
```

```
  z = log10(badluckdata[,2])),
  labels = badluckdata[,6],
  cex = 0.4, adj = -0.2, col="black")

my.lm <- lm(log10(badluckdata[,2]) ~ log10(badluckdata[,3]) +
              log10((u * badluckdata[,4])^drivers))
s3d$plane3d(my.lm, col="grey")
```

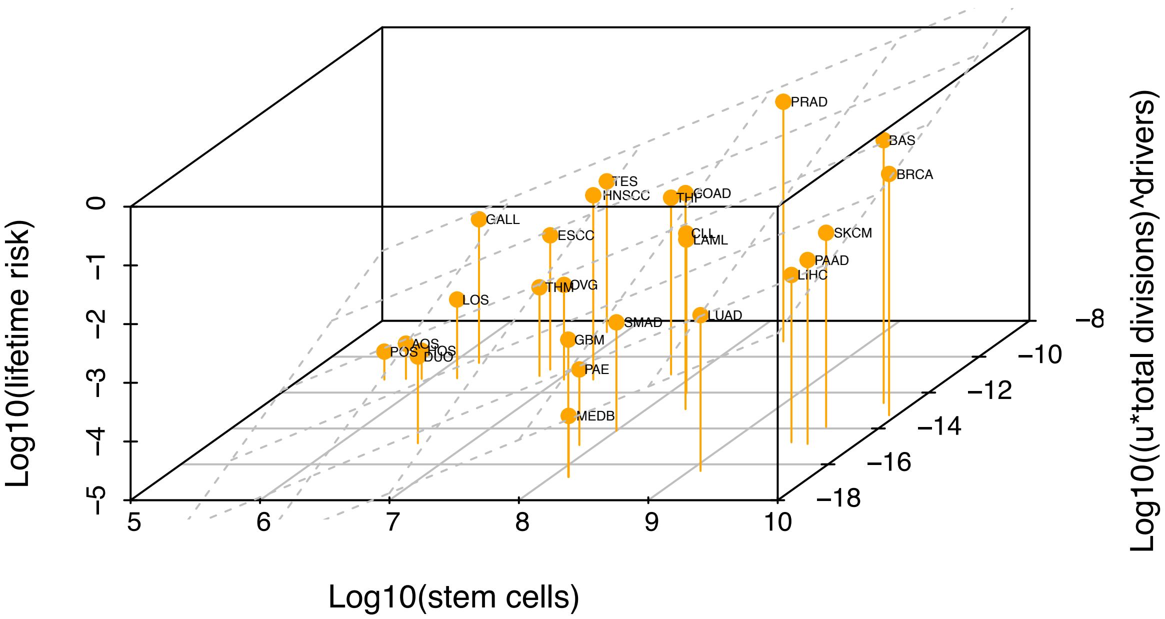


```
my.lm
```

```
##
## Call:
## lm(formula = log10(badluckdata[, 2]) ~ log10(badluckdata[, 3]) +
##     log10((u * badluckdata[, 4])^drivers))
##
## Coefficients:
##                           (Intercept)                log10(badluckdata[, 3])
##                               -6.0359                                 0.9070
## log10((u * badluckdata[, 4])^drivers)
##                                0.2885
```

S6.4 Sensitivity analysis

We check for the robustness of the results via one-factor-at-a-time (OAT) by letting the number of drivers in each cancer type be a random variable. Specifically, a discrete uniform distribution over {2,3,4} for cancer types estimated to have 3 drivers, {1,2,3} for cancer types estimated to have 2 drivers, and {1,2} for cancer types estimated to have only one driver. The sensitivity analysis for the number of cells and division rates was already performed in Tomasetti and Vogelstein 2015 (Variation in cancer risk among tissues can be explained by the number of stem cell divisions. Science 2015, 347(6217):78-81).

```
set.seed(123)
matrix_coef <- matrix(0, nrow = 10000, ncol = 4)

for (i in 1:10000) {
    Drivers <- replicate(length(badluckdata[, 5]), 0)
```

```r
    Drivers[badluckdata[, 5] == 1] <- round(runif(length(badluckdata[,
        5] == 1), min = 1, max = 2))
    Drivers[badluckdata[, 5] == 2] <- round(runif(length(badluckdata[,
        5] == 2), min = 1, max = 3))
    Drivers[badluckdata[, 5] == 3] <- round(runif(length(badluckdata[,
        5] == 3), min = 2, max = 4))
    Drivers[badluckdata[, 5] == 4] <- round(runif(length(badluckdata[,
        5] == 4), min = 3, max = 5))

    # we keep all but one of the original values
    nochange <- sample(1:26, 25, replace = FALSE)

    Drivers[nochange] <- badluckdata[, 5][nochange]

    Drivers[5] <- Drivers[1]
    Drivers[c(12, 14, 21)] <- Drivers[2]
    Drivers[c(7, 23)] <- Drivers[6]

    linereg = lm(log10(badluckdata[, 2]) ~ log10(badluckdata[,
        3]) + log10((u * badluckdata[, 4])^(Drivers)))

    matrix_coef[i, ] <- c(linereg$coefficients, summary(linereg)$adj.r.squared)
}


mean(matrix_coef[, 2])
```

```
## [1] 0.8861895
```

```r
mean(matrix_coef[, 3])
```

```
## [1] 0.265785
```

```r
mean(matrix_coef[, 4])
```

```
## [1] 0.7755626
```

```r
sd(matrix_coef[, 2])
```

```
## [1] 0.04320113
```

```r
sd(matrix_coef[, 3])
```

```
## [1] 0.03577291
```

```r
sd(matrix_coef[, 4])
```

```
## [1] 0.03853435
```

References

[1] Charles, D. R. & Luce-Clausen, E. M. The kinetics of papilloma formation in benzpyrene-treated mice. *Cancer Res.* **2**, 261–263 (1942).

[2] Frank, S. A. Dynamics of cancer: incidence, inheritance, and evolution. (Princeton University Press, 2007).

[3] Nordling, C. O. A new theory on cancer-inducing mechanism. *Br. J. Cancer* **7**, 68–72 (1953).

[4] Arley, N. & Iversen, S. On the mechanism of experimental carcinogenesis. *Acta Pathol Microbiol Scand* **31**, 164-171 (1952).

[5] Armitage, P. & Doll, R. The age distribution of cancer and a multi-stage theory of carcinogenesis. *Br. J. Cancer* **8**, 1–12 (1954).

[6] Armitage, P. & Doll, R. A two-stage theory of carcinogenesis in relation to the age distribution of human cancer. *Br. J. Cancer* **11**, 161–169 (1957).

[7] Moolgavkar, S. H. & Venzon, D. J. Two-event models for carcinogenesis: incidence curves for childhood and adult tumors. *Math. Biosci.* **47**, 55–77 (1979).

[8] Moolgavkar, S. H. & Knudson, A. G. Mutation and cancer: a model for human carcinogenesis. *J. Natl. Cancer. Inst.* **66**, 1037-1052 (1981).

[9] Moolgavkar, S. H. & Luebeck, G. E. Two-event model for carcinogenesis: biological, mathematical, and statistical considerations. *Risk Anal.* **10**, 323-41 (1990).

[10] Heidenreich, W. F. & Paretzke, H. G. The two-stage clonal expansion model as an example of a biologically based model of radiation-induced cancer. *Radiat. Res.* **156**, 678–681 (2001).

[11] Durrett, R. & Moseley, S. Evolution of resistance and progression to disease during clonal expansion of cancer. *Theor. Popul. Biol.* **77**, 42–48 (2010).

[12] Tomasetti, C., Vogelstein, B. & Parmigiani, G. Half or more of the somatic mutations in cancers of self-renewing tissues originate prior to tumor initiation. *PNAS* **110**, 1999–2004 (2013).

[13] Dutta, A., Dutreux, F. & Schacherer, J. Loss of heterozygosity results in rapid but variable genome homogenization across yeast genetic backgrounds. *eLife* **10**, e70339 (2021).

[14] Tomasetti, C., Marchionni, L., Nowak, M. A., Parmigiani, G. & Vogelstein, B. Only three driver gene mutations are required for the development of lung and colorectal cancers. *PNAS* **112**, 118–123 (2015).

[15] Iranzo, J., Martincorena, I. & Koonin, E. V. Cancer-mutation network and the number and specificity of driver mutations. *PNAS* **115**, E6010–E6019 (2018).

[16] Tomasetti, C. & Vogelstein, B. Variation in cancer risk among tissues can be explained by the number of stem cell divisions. *Science* **347**, 78–81 (2015).

[17] Tomasetti, C., Li, L. & Vogelstein, B. Stem cell divisions, somatic mutations, cancer etiology, and cancer prevention. *Science* **355**, 1330–1334 (2017).
