# Supplementary material for: Evaluating cancer etiology and risk with a mathematical model of tumor evolution
Source: Nat Commun. 2022 Nov 24;13:7224. doi: 10.1038/s41467-022-34760-1 (PMC9700699; doi:10.1038/s41467-022-34760-1)
Supplement: Supplementary file 3 — Description of Additional Supplementary Files [file 41467_2022_34760_MOESM3_ESM.pdf]

**Description of Additional Supplementary Files**

**Supplementary Software**: A code implementing formula (2) in the Wolfram Language.
