# Supplementary material for: Evaluating cancer etiology and risk with a mathematical model of tumor evolution
Source: Nat Commun. 2022 Nov 24;13:7224. doi: 10.1038/s41467-022-34760-1 (PMC9700699; doi:10.1038/s41467-022-34760-1)
Supplement: Supplementary file 4 — Supplementary Software [file 41467_2022_34760_MOESM4_ESM.zip › SupplementarySoftware/readme.rtf]

ExpectedNbMutationsThe file ExpectedNbMutations.nb is a Wolfram notebook. System requirements and installationIt must be opened with the software Wolfram Mathematica and has been tested on version 12.0.0.0. No install time is required.OutputThe function expected[mu, c, n, init, b, bc, rho, a] provides as an output the expected number of mutations in a cancer cell lineage of a patient of age a for a given tissue and cancer type with parameters:- n = number of required drivers- init = number of divisions that occur during development:- b = division rate of the healthy tissue- bc = division rate in cancer tissue- rho = proportion of the population that gets that cancer by age 80,assuming that the background mutation rate is mu and that cancer occurs between time a-c and c.DemoThe dataset DemoDataset.xlsx provides in the second column the output of expected[0.03, 5, 3, 33, 4.325, 73, 0.0107][a], where a is in the first column (these parameters correspond to the cancer type BRCA as given in Table 1).The run time is less than a few seconds.
